# Twenty questions from the surgeon to the radiologist to better plan an open partial horizontal laryngectomy

**DOI:** 10.3389/fonc.2023.1305889

**Published:** 2024-01-24

**Authors:** Erika Crosetti, Giovanni Succo, Silvia Sapino, Ilaria Bertotto, Stefano Cirillo, Massimo Petracchini, Gabriele Fondello, Giulia Arrigoni, Martina Tascone, Cesare Piazza, Davide Farina, Marco Ravanelli

**Affiliations:** ^1^ Head and Neck Cancer Unit, San Giovanni Bosco Hospital, Turin, Italy; ^2^ Oncology Department, University of Turin, Head and Neck Cancer Unit, San Giovanni Bosco Hospital, Turin, Italy; ^3^ Radiology Service, Candiolo Cancer Institute, Candiolo, Italy; ^4^ Radiology Service, Mauriziano Umberto I Hospital, Turin, Italy; ^5^ Department of Otorhinolaryngology—Head and Neck Surgery, ASST Spedali Civili di Brescia, University of Brescia, School of Medicine, Brescia, Italy; ^6^ Department of Radiology, University of Brescia, Brescia, Italy

**Keywords:** laryngeal cancer, partial laryngectomy, OPHL, imaging, magnetic resonance imaging, computerized tomography

## Abstract

Open partial horizontal laryngectomies (OPHLs) represent a valuable therapeutic option for tumors of the intermediate T-category and, in selected cases, for locally advanced tumors with low-volume extra-laryngeal extension. The eligibility of patients treated with this type of surgery has increased with the introduction of the modular approach to OPHL planning. This strategy follows the introduction of the classification proposed by the European Laryngological Society, based on the extent of horizontal resection. Optimization of the selection is the result of a meticulous work-up process involving close cooperation between experienced surgeons and radiologists, followed by final quality control by pathologists. Computed tomography and magnetic resonance imaging are study methods whose pearls and pitfalls are well known, especially when performed at a high level of expertise. In this paper, based on the experience of two high-volume centers, a checklist of 20 questions addressed by the surgeon to the radiologist before planning an OPHL was proposed. Considerations regarding case selection are reported for each of the questioned parameters. A very simple question-and-answer process is easy to understand and mainly addressed by less experienced colleagues who wish to increase their knowledge and skills in performing this type of surgery.

## 1 Introduction

Open partial surgery of the larynx represents an important tool in the hands of the multidisciplinary tumor board for the conservative treatment of intermediate-advanced T-categories of laryngeal cancer (LC). Considering the high percentage of patients suffering from such a disease, partial surgery in healthy margins corresponds to a high potential for loco-regional control, an excellent chance of long overall/disease-specific survival with a single treatment, and equally high chances of preserving good laryngeal functions (1).

Prerequisites for the success of this treatment are a careful selection of candidates (based on the patient’s general condition) (2), as well as a thorough selection of radically resectable tumors using an open partial laryngectomy approach (3). In fact, these operations differ in the amount of tissue and laryngeal structures removed, which is directly linked to the different modalities of functional reconstruction of the residual larynx.

In the field of open partial laryngeal surgery, a real revolution has ensued from the introduction in 2014 of the European Laryngological Society (ELS) classification of open partial horizontal laryngectomies (OPHL) (4). This classification, based on the lower limit of resection to be accomplished (type I → supraglottic, type II → supracricoid, type III → supratracheal), has in fact paved the way for a “renaissance” of this surgery, essentially linked to the modulation of the 12 different types of operation that make up the OPHL system framework, aimed at achieving a safe surgical radicality checked by the pathologist.

Therefore, having a surgical solution that can be modulated according to the extent of the disease and considering the excellent oncological/functional results reported on patients selected for general characteristics, it can be stated that most of the surgical planning derives from an accurate preoperative diagnostic work-up (5, 6). This process, based on clinical, endoscopic, and imaging data, must review anatomically and functionally, negatively affecting the primary goal of any form of surgery, i.e., the achievement of radicality and, in this specific case, also the equally important goal of functional reconstruction of the residual organ.

Therefore, the aim of this study was to present a list of 20 precise questions that the surgeon usually asks to radiologist when planning an OPHL operation. For each of the questiosn and answers, based on the experience of the authors, the main effects on pre- and intraoperative decision-making concerning OPHL interventions have been reported and depicted iconographically.

## 2 Methods

A review of the main published articles on this topic was carried out and subsequently compared with the experiences of two high-volume tertiary referral centers for laryngeal cancer. The result of this process is a series of precise questions that the clinician asks the radiologist in daily practice, which are followed by equally precise answers supported by rich explanatory iconography. All are presented a guide for the approach to this type of surgery.

### 2.1 General rules for preoperative diagnostic work-up

**Figure 1:**
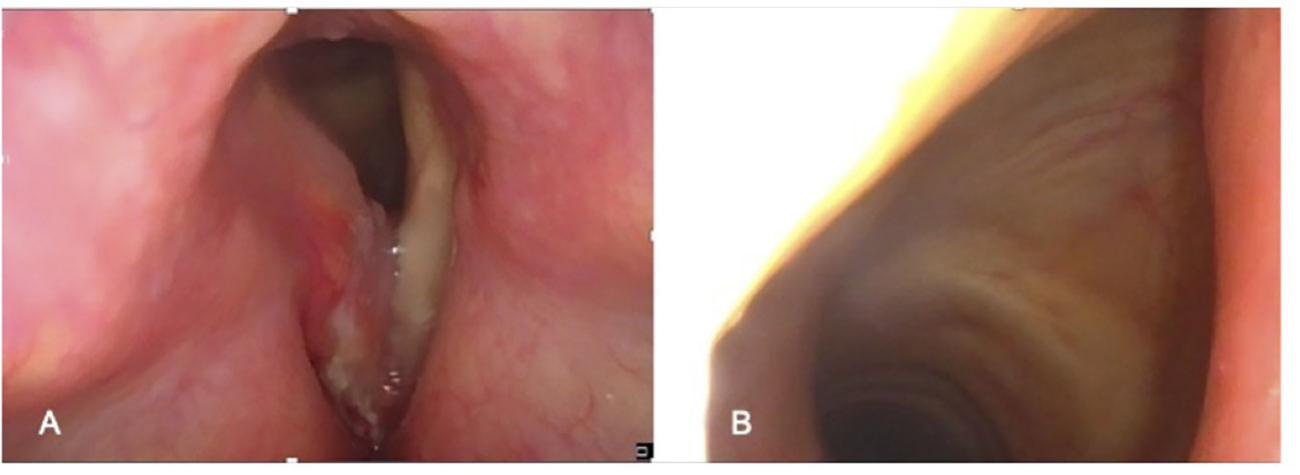
Preoperative endoscopic workup in the office: **(A)** Endoscopic view obtained with clinician positioned in front of the patient. **(B)** Endoscopic view obtained with clinician positioned behind the patient (better visualization of the subglottis).

The process of selecting cases that can be treated by OPHL always begins with state-of-the-art videoendoscopic examination. It is preferable to perform this examination with the patient semi-seated and the operator placed behind him after careful contact anesthesia of the upper aero-digestive tract mucosa. The subglottic site could be better assessed at this position (**Figures 1A, B**). The aim of preoperative endoscopy is to assess three important parameters: (A) superficial extent of the disease, (B) potential submucosal extension, and (C) vocal cord and arytenoid motility.

Imaging is always performed before biopsy under direct microlaryngoscopy, in which parameters A and B are checked even more accurately. Currently, contrast-enhanced multidetector computed tomography (CT) is the first-line technique for LC assessment. A short acquisition time reduces motion artifacts; furthermore, CT can be performed using dynamic maneuvers (inspiration, “E” phonation, Valsalva). Multiplanar reconstructions (i.e., axial plane parallel to the glottis, coronal, or sagittal) allow the detection of tumor spread on different planes (6, 7). Nevertheless, limitations of CT imaging are widely known and may be summarized in its low sensitivity, especially when cartilaginous tumor invasion or extra- laryngeal spread must be assessed (8, 9).

Magnetic resonance imaging (MRI) is characterized by intrinsically high contrast resolution, which permits better tissue discrimination and more sophisticated anatomical details. Diffusion-weighted imaging (DWI) improves the ability of MRI to detect small lesions and to discriminate the tumor from peritumoral inflammation. However, acquisition times that are longer than those of CT result in an increased risk of motion artifacts. Therefore, optimized scanning protocols (e.g., radial k-space filling sequences, which offer partial compensation for involuntary motion) and adequate patient instructions are essential ingredients for a high-quality scan (5, 7, 8). MRI is considered preferable to CT for LC staging when organ preservation surgery (i.e., OPHL) is planned and in cases of recurrence after radiation (RT) or transoral laser microsurgery (TOLMS) (7).

### 2.2 List of questions to be made to the radiologist before an OPHL

#### 2.2.1 Is there extra-laryngeal spreading into the base of the tongue and/or piriform sinus?

**Figure 2:**
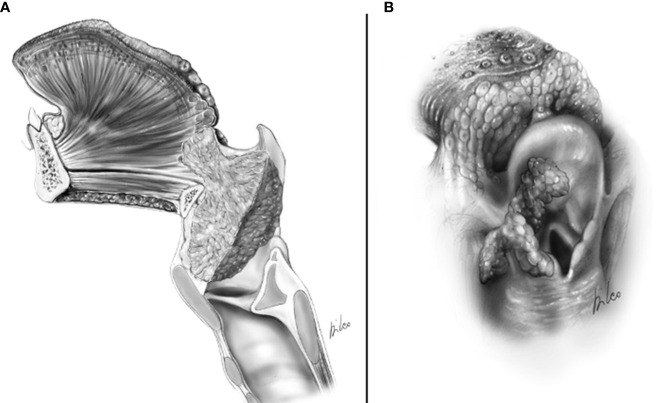
cT4a supraglottic tumor: **(A)** Neoplastic spreading to the glosso-epiglottic vallecula and base of tongue. **(B)** Neoplastic spreading to the lateral wall of the piriform sinus.

The precise assessment of the base of the tongue (BOT) involvement by a supraglottic tumor is of paramount importance because it represents an absolute contraindication to OPHL types II and III and a relative contraindication to OPHL type I (10). Imaging can demonstrate with great accuracy this feature that shifts tumor to cT4a, but, at the same time, it may be difficult to be appreciated on clinical examination in case of very bulky lesions of the suprahyoid epiglottis. In fact, such tumors may either directly affect the mucosa of the vallecula and BOT by infiltrating the intrinsic musculature of the tongue or reach and infiltrate the extrinsic musculature of the tongue after massive involvement of the pre-epiglottic space (PES), eventually protruding into the vallecular submucosa (**Figure 2A**).

Conventionally, involvement of more than 2 cm of the intrinsic musculature at the level of the BOT is a contraindication to OPHL type I + BOT due to the absence of a protective effect on the airway determined by the retraction of the tongue base overlying the glottis (11).

Laterally, the tumor of the aryepiglottic fold may extend to the pharyngoepiglottic fold and lateral wall of the piriform sinus (**Figure 2B**). This feature also represents an absolute contraindication to OPHLs types II and III and a relative contraindication to OPHL type I + PIR. In the latter case, it becomes an absolute contraindication when the extension to the lateral wall of the piriform sinus becomes massive, affecting the lateral-posterior wall of the hypopharynx.

**Figure 3:**
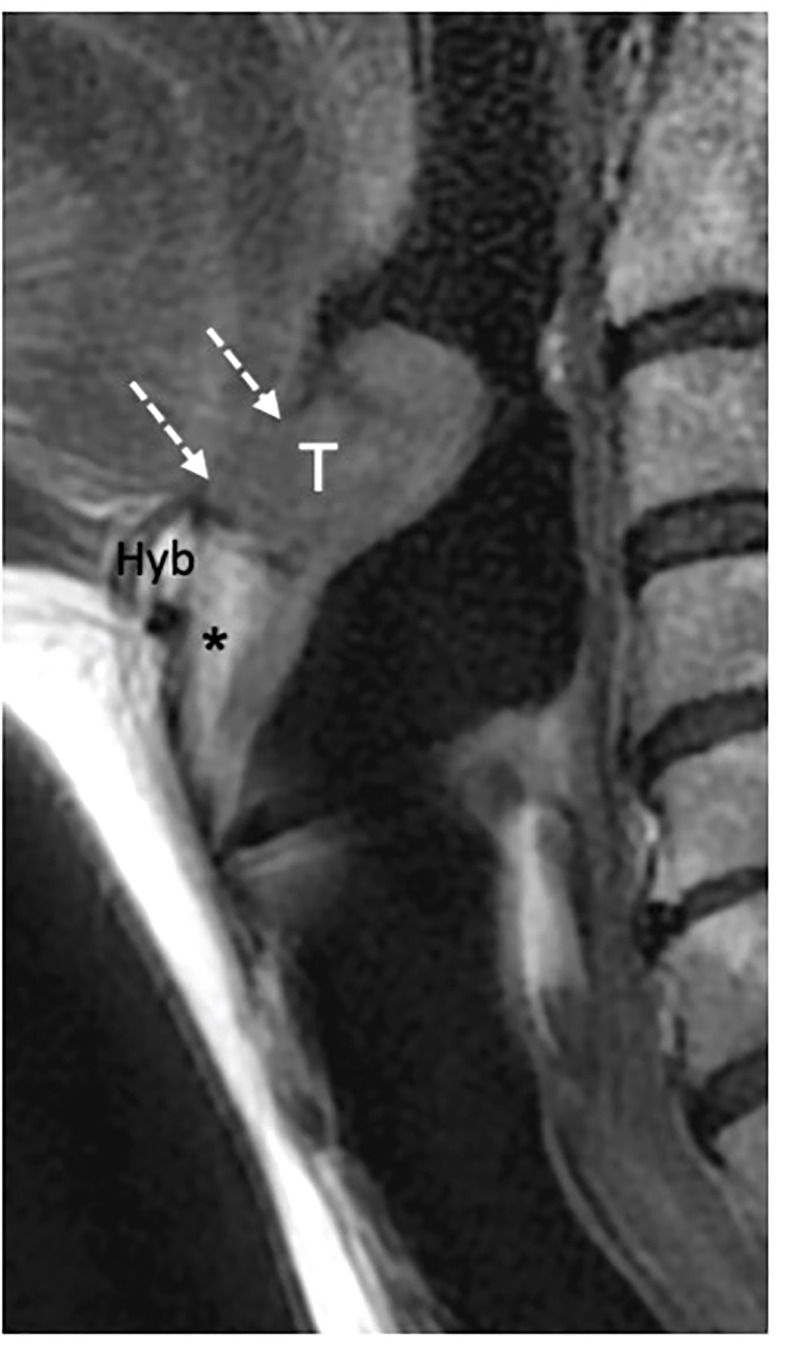
MRI T2-weighted image on sagittal piane shows a supraglottic tumor (T) involving the linguai surface of the suprahyoid epiglottis. The lesion is adjacent to the base of tongue (dashed white arrows) that is not involved. Hyoid bone (Hyb) is normal as well as the pre- epiglottic space (asterisk).

CT and MRI revealed tumor invasion into the BOT. The depth of invasion was measured more accurately in the sagittal plane (**Figure 3**). Tumor extension to the BOT should not be confused with lingual tonsil hypertrophy. On both CT and MRI, careful scrutiny of the tissue pattern (described as “columnar” or “striated”) and contours (lobulated) generally permits to correctly identify lingual tonsil hypertrophy, even if slightly asymmetric. Notably, DWI was not helpful in this case because both the tumor and the normal lingual tonsil displayed diffusion restriction.

The piriform sinus often collapses and is consequently, poorly evaluated on imaging. The fast scanning time of CT offers the possibility of integrating the exam with an acquisition performed during the Valsalva maneuver, which expands the sinus itself. On MRI this is generally not possible; however, the combination of conventional and DWI sequences may adequately demonstrate submucosal neoplastic spread to this site in most cases (12).

#### 2.2.2 Is the hyoid bone involved?

It must be considered that every type of OPHL involves preservation of the hyoid bone, which is the anchoring structure of the pexy, allowing anatomical and functional stabilization of the neolarynx. Therefore, precise preoperative knowledge of the possible neoplastic involvement of the hyoid bone is of utmost importance, although it is quite rare.

Kirchner and Ogura reported a specimen analysis of supraglottic tumors without finding signs of invasion of the hyoid bone, while Timon et al. described a very low percentage of focal hyoid invasion (1.4%) caused mainly by supraglottic tumors originating from the aryepiglottic fold or vallecular tumors (13–15). Most neoplastic infiltrations occur in the hyoid great horn or at the junction between the hyoid body and its horns. The reason for this low incidence of hyoid bone invasion has been well explained by Kirchner’s pathological studies of whole-organ sections (16). The front of the tumor growth, in fact, due to confinement by the fibroelastic membrane of the thyro-epiglottic ligament and/or thyrohyoid membrane, is isolated from direct contact with the hyoid bone.

The confirmation of any hyoid involvement on imaging contraindicates the performance of every type of OPHLs and must be clinically suspected and addressed by the radiologist whenever a bulky submucosal lesion of the vallecula is seen.

**Figure 4:**
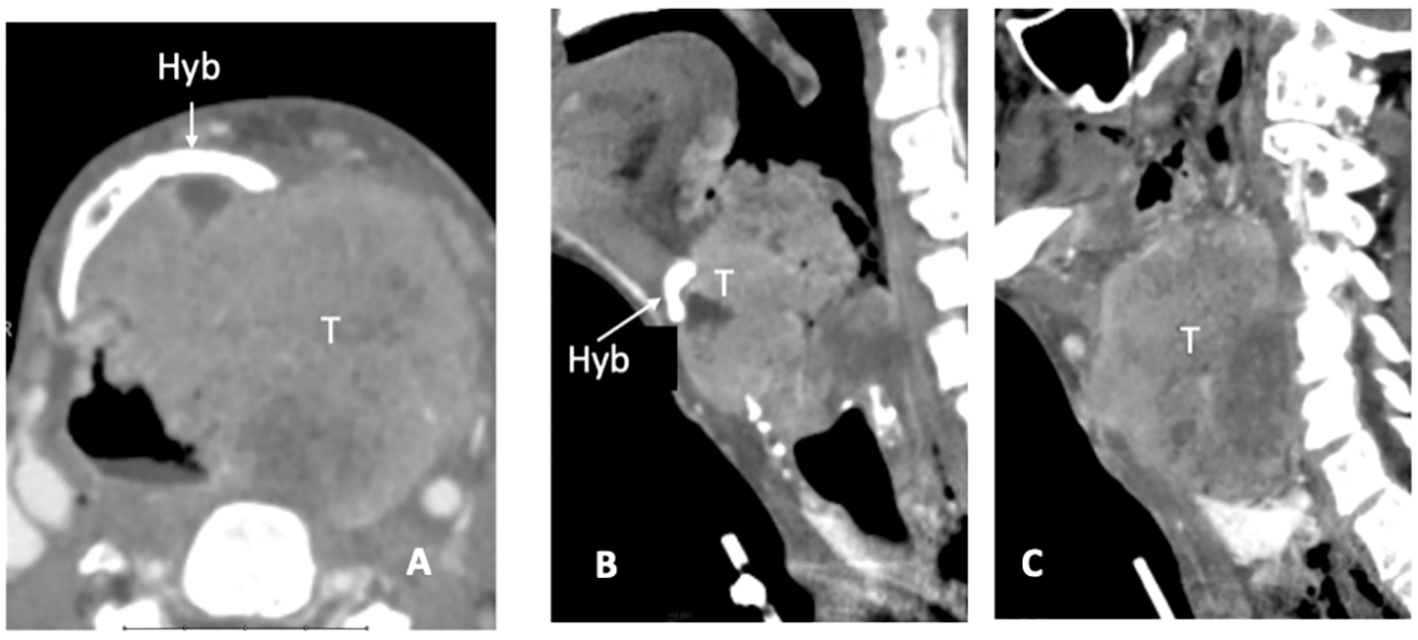
CT scan, axial plane **(A)**, at the supraglottic level, a bulky laryngeal cancer (T) invades the left side of the hyoid bone (Hyb) and presents massive extra-laryngeal spread. On sagittal plane **(B)** the median part of the hyoid bone is detectable (white arrow), while in the second (left paramedian) image the left portion is replaced by tumour tissue **(C)**.

CT detects subtle cortical bone erosion. However, MRI is superior for assessing the medullary bone. On plain T1, the replacement of bright fat tissue with a hypointense signal is a non-specific indicator of abnormality; DWI and contrast-enhanced imaging help refine the diagnosis and discriminate tumor invasion from edema. Owing to its spatial orientation, the hyoid was best assessed on the axial and sagittal planes (**Figures 4A–C**).

#### 2.2.3 Does the tumor pass through the epiglottis and involve the pre-epiglottic space?

This information is of utmost importance in planning all kinds of OPHL, particularly types IIa and IIIa, which provide access to the laryngeal vestibule through trans-epiglottic laryngotomy. In OPHL types I, IIb, and IIIb, en bloc resection of the PES adipose content is always performed.

PES involvement, especially in the early phases, can only be detected radiologically. Because of the high adipose tissue content and its median position, this space can be easily evaluated both on multiplanar CT and MRI and better highlighted in the sagittal and axial planes (17). The sagittal plane is particularly helpful when assessing the marginal sovracommissural extent. The axial plane is best suited to depict tumor growth in the lateral parts of the PES and from there (with no anatomical barrier) to the superior paraglottic space (PGS).

**Figure 5:**
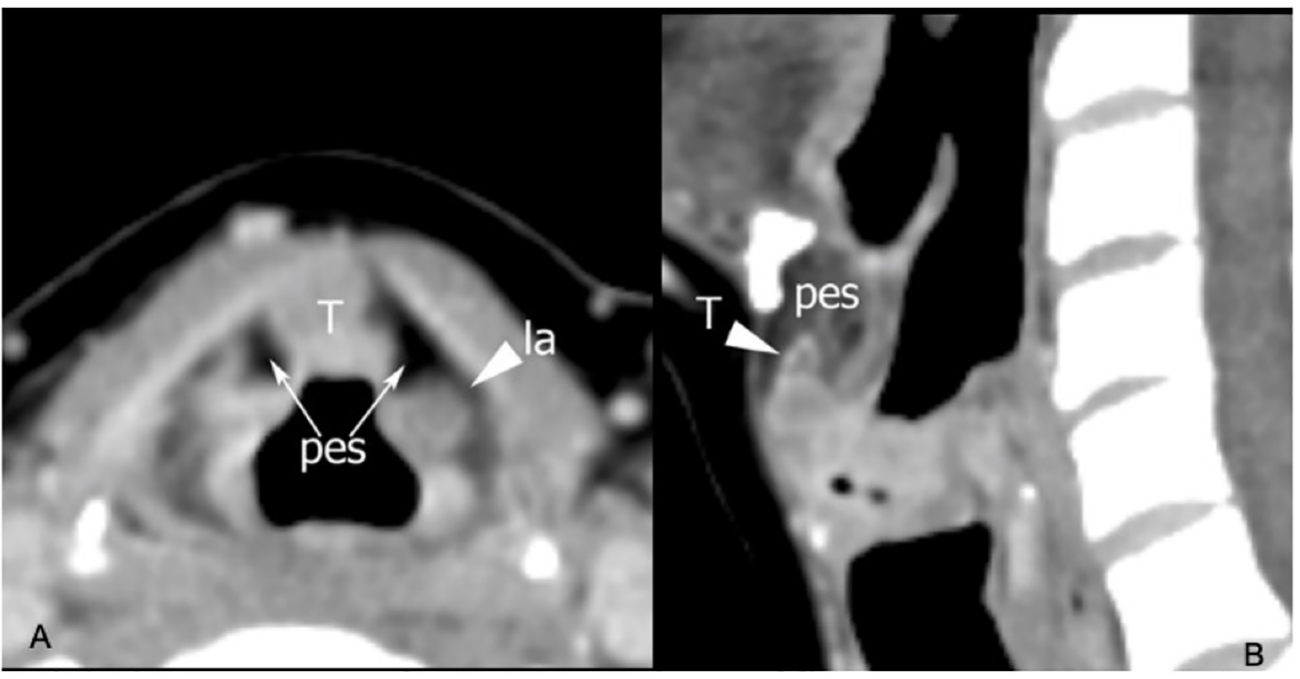
CT on axial plane **(A)** at the supraglottic level identifies tumoral tissue (T) involving the median part of the pre-epiglottic space (PES), while the lateral portions of this visceral space (arrows) show regular fat density. Arrowhead points a laryngocele (la) in the left superior paraglottic space. On sagittal plane **(B)** the lesion invades the inferior part of the PES.

**Figure 6:**
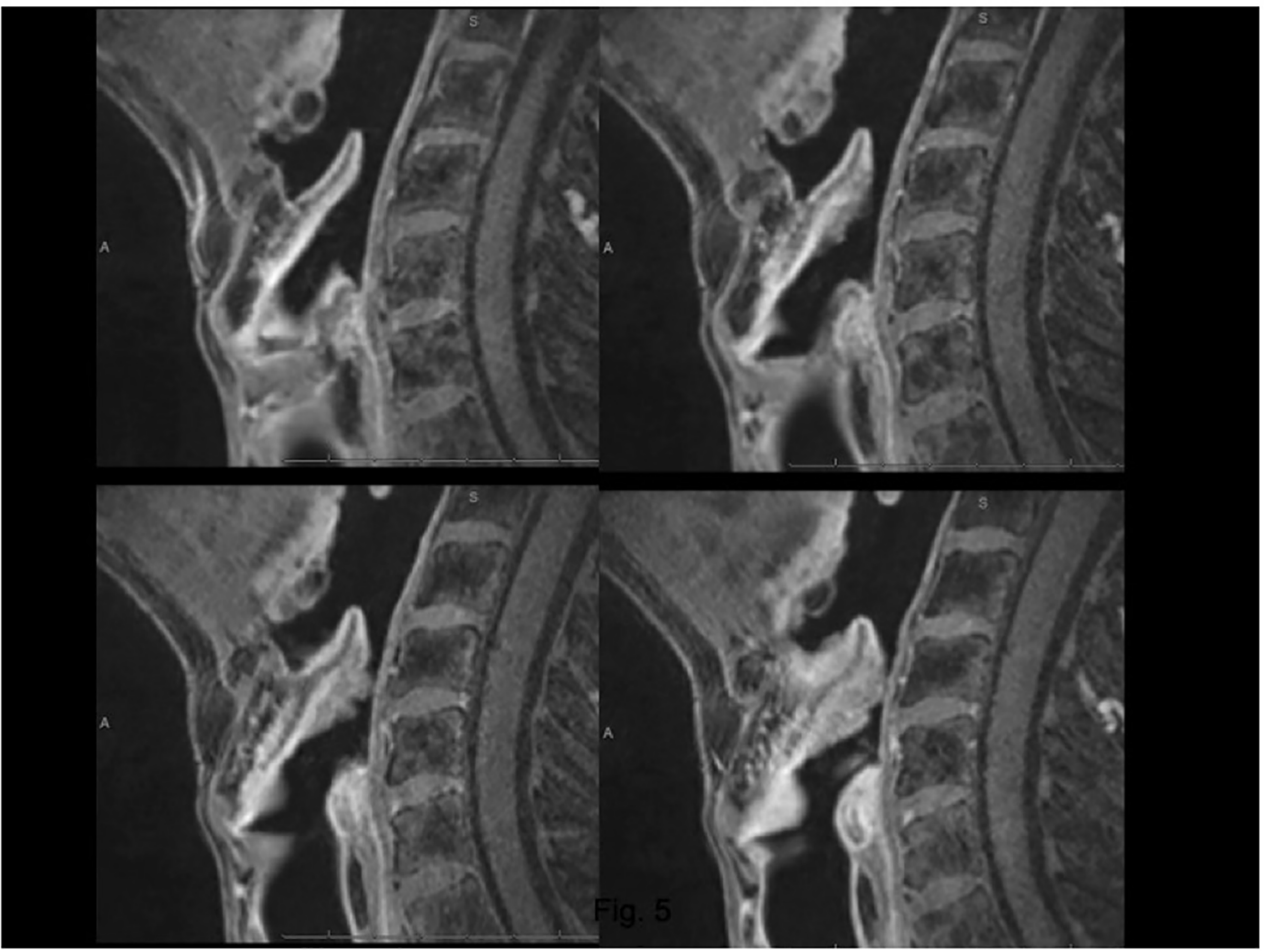
MRI, T1 fat-sat sequence (VIBE) on sagittal plane. Lesion arising from the epiglottis, which infiltrates both its supra- and infrahyoid portions, up to the petiole. Effacement of the posterior part of the median pre-epiglottic space.

The clinical–endoscopic examination may highlight an infiltrative attitude of the tumor in areas at greater risk of extension to the PES, such as the infra-petiolar region and junction of the ventricular bands with the epiglottis (**Figures 5A, B**, **6**).

In terms of surgical choices, the PES involvement contraindicates the performance of any trans-epiglottic laryngotomy that divides the fat content of the PES. However, this maneuver is only envisaged for OPHL types IIa and IIIa.

**Figure 7:**
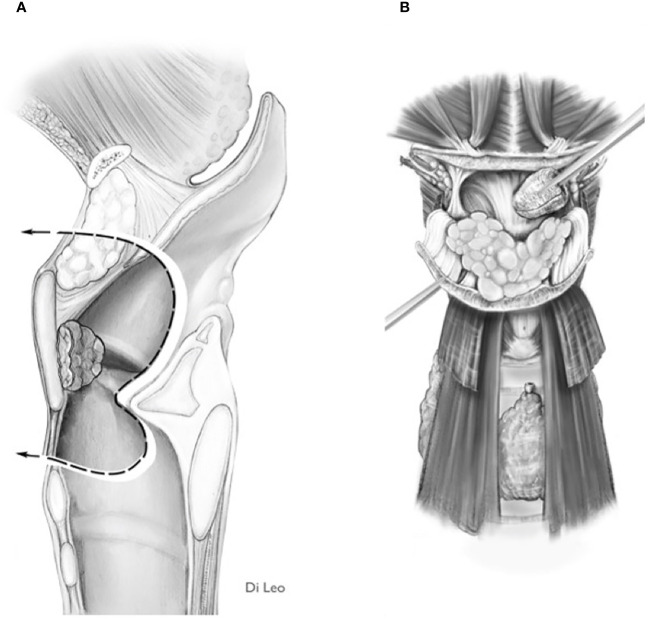
Scheme of a Type Ila OPHL resection: pre-epiglottic space (PES) management. **(A)** superior access by a trans-epiglottic laryngotomy through thè PES. **(B)** the PES content is dissected and pulled down, before proceeding with thè trans-epiglottic laryngotomy (variant).

If the imaging information shows such involvement, the surgeon is faced with two options: 1) Carry out trans-epiglottic laryngotomy only after completely dissecting and reversing the content of the PES (**Figures 7A, B**).

**Figure 8:**
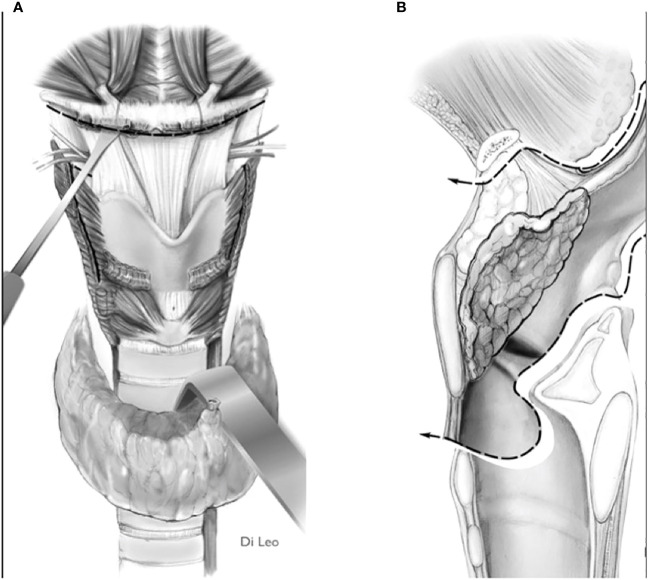
Scheme of a type llb OPHL resection: the whole pre-epiglottic space is included in the specimen. **(A)** frontal view. **(B)** sagittal view.

2) Convert the indication from a type IIa/IIIa OPHL to a type IIb/IIIb OPHL, which is certainly a more radical solution because it involves complete en bloc removal of the PES with the associated supraglottic structures (**Figures 8A, B**).

#### 2.2.4 Does the tumor pass through the thyro-hyoid membrane?

**Figure 9:**
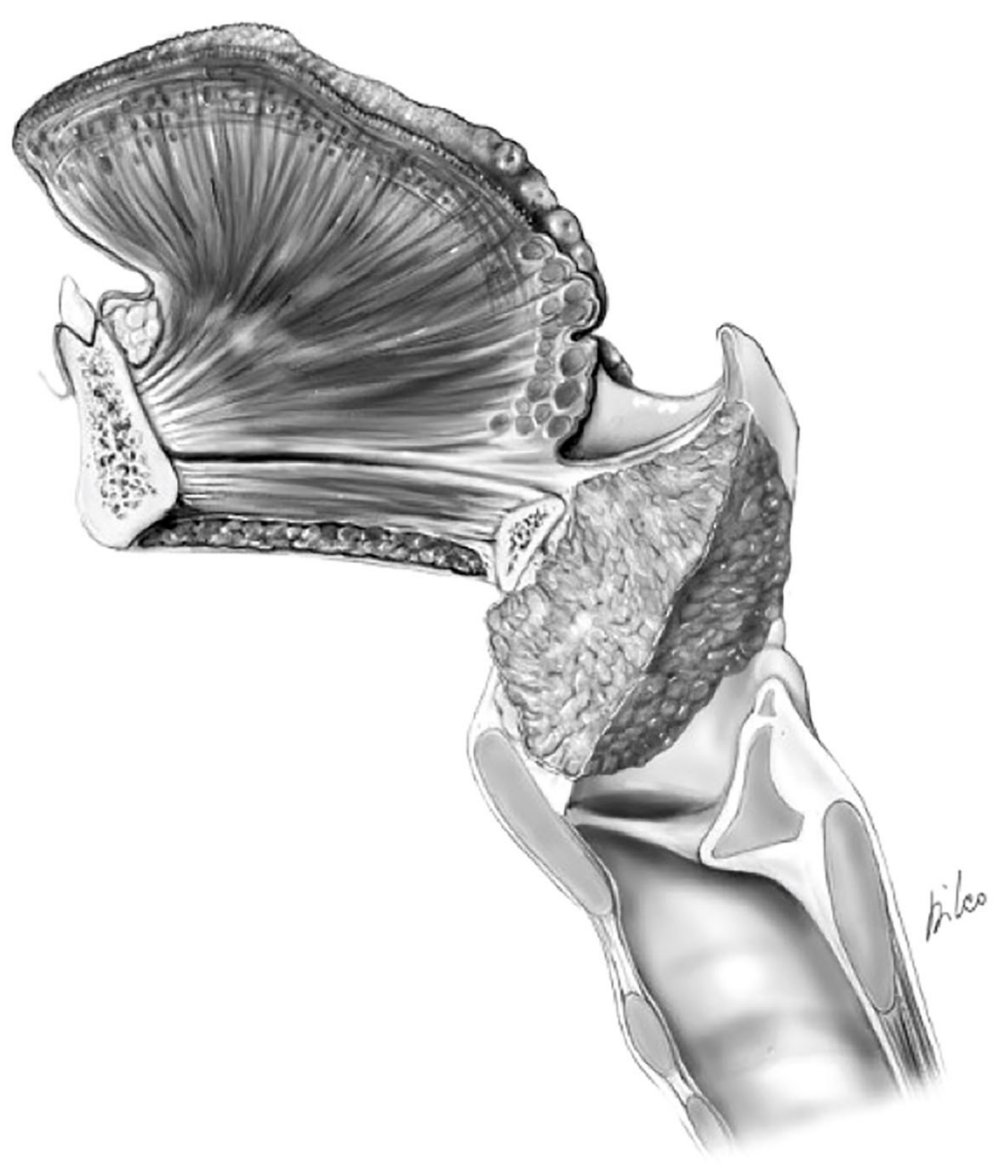
cT4a supraglottic tumor: minimal spreading outside the larynx through the thyro-hyoid membrane.

Correct assessment of this aspect is crucial for the indication or contraindication of OPHL. Supraglottic tumors can extend to the prelaryngeal tissues through the thyrohyoid membrane and its holes for the passage of the superior laryngeal neurovascular pedicles (11, 13, 15) (**Figure 9**).

**Figure 10:**
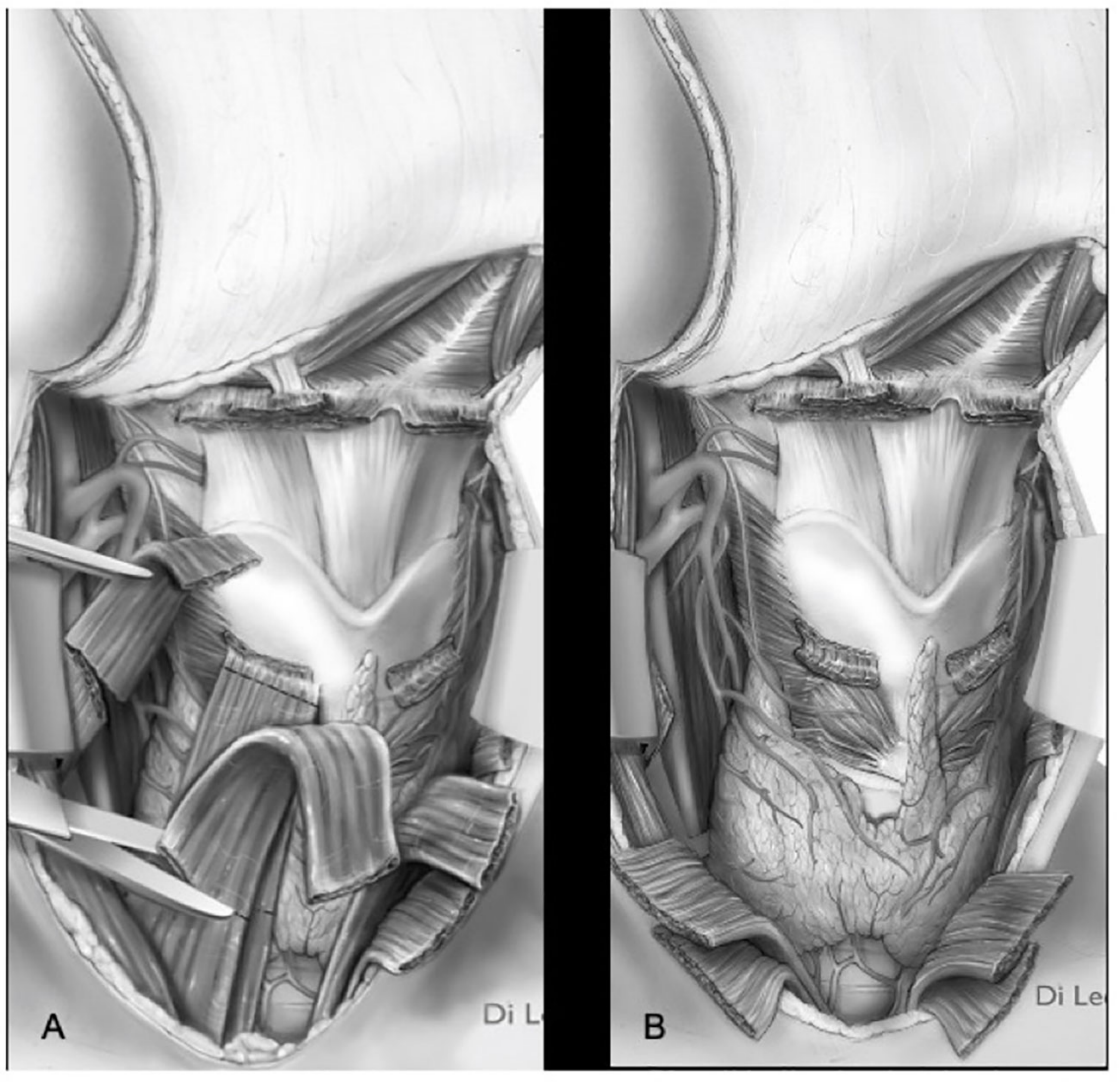
Types ll–lll OPHL with strap muscles resection **(A)** section of strap muscles. **(B)** strap muscles resected.

Itamura et al. have shown how cases in which the tumor may bulge the membrane without frank invasion of the prelaryngeal tissues can be differentiated in terms of both prognostic and therapeutic outcomes in cases in which there is clear extension through the membrane and gross extra-laryngeal extension (18). In a recent publication, Succo et al. demonstrated good functional results for low-volume extra-laryngeal pT4a tumors, with minimal or absent cartilage destruction, treated with OPHLs. In selected cases, i.e., clinically and radiologically suspected for minimal extra-laryngeal extension, OPHLs, potentially providing whole strap muscle resection (**Figures 10A, B**), can guarantee the same anterior radicality as a total laryngectomy (19).

**Figure 11:**
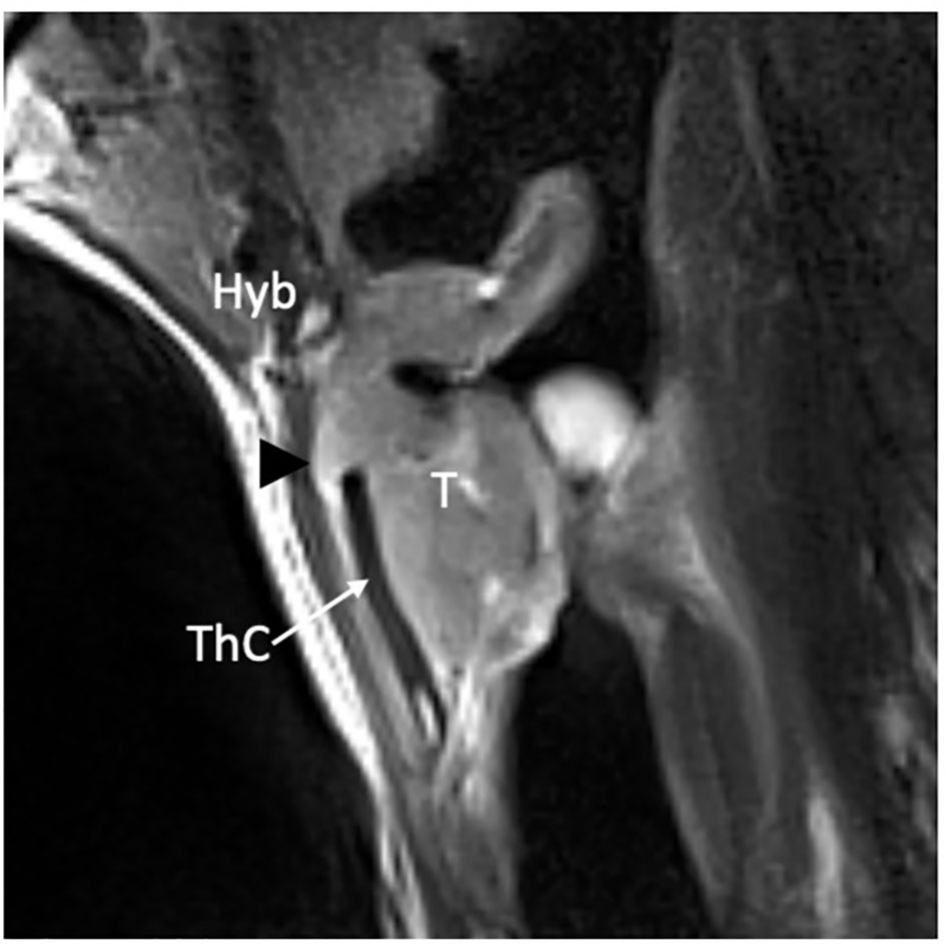
MRI T2-weighted image on parasagittal plane demonstrates a supraglottic lesion (T) that reaches the thyroid notch (black arrowhead) with suspected infiltration of the thyrohyoid membrane above the superior margin of a non-ossified thyroid lamina (ThC) that shows regular signal intensity as the hyoid bone (Hyb).

MRI has improved the evaluation accuracy of this rare form of extra-laryngeal extension, which can occur even in the absence of erosion of the upper border of the thyroid cartilage. A study by Chen et al. showed that only 44% of pathologically proven extra-laryngeal diseases had thyroid cartilage penetration. Furthermore, CT demonstrated low sensitivity in the identification of extra-laryngeal spread (49%) (20). A trick for the radiologist is to obtain parasagittal images perpendicular to the thyroid laminae, which can be made with multiplanar reconstruction after volumetric image acquisition (both in CT and MRI) or directly acquiring images on this plane (in MRI). The thyrohyoid membrane is not directly visualized; however, sharp margins at the tumor and soft-tissue interface suggest bulging on the membrane without penetration (**Figure 11**).

In the case of mis-under/over-staging of these borderline cT3–cT4a tumors, OPHL type IIb, which involves resection of the entire thyroid cartilage in addition to the thyroid membrane and strap muscles, seems to provide a sufficient radicality.

#### 2.2.5 Is the superior paraglottic space involved?

Generally, supraglottic tumors that originate or involve the false cord and aryepiglottic fold tend to invade the superior PGS and extend inferiorly to cross the laryngeal ventricle, thus becoming ‘transglottic’ (17, 21). The assessment of superior PGS by CT and MRI needs to be performed on the axial and coronal planes (17).

Precisely knowing the extent of this possible progression favors the correct planning of an OPHL. Indeed, the absence of anatomical barriers between the adipose tissue of the superior PGS and the PES and the possibility that the tumor affects the submucosa of the medial wall of the piriform sinus, the arytenoid, and the inferior PGS impose serious considerations before indicating an OPHL and its type (16).

Several details must be studied during the preoperative workup:

A) Involvement of the aryepiglottic fold, which often occurs in a submucosal mode, resulting in its enlargement in relation to the contralateral one. On imaging, this is a relatively easy diagnosis, which can be obtained by simply matching the thickness and signal/density of the fat tissue content of the paired folds.

**Figure 12:**
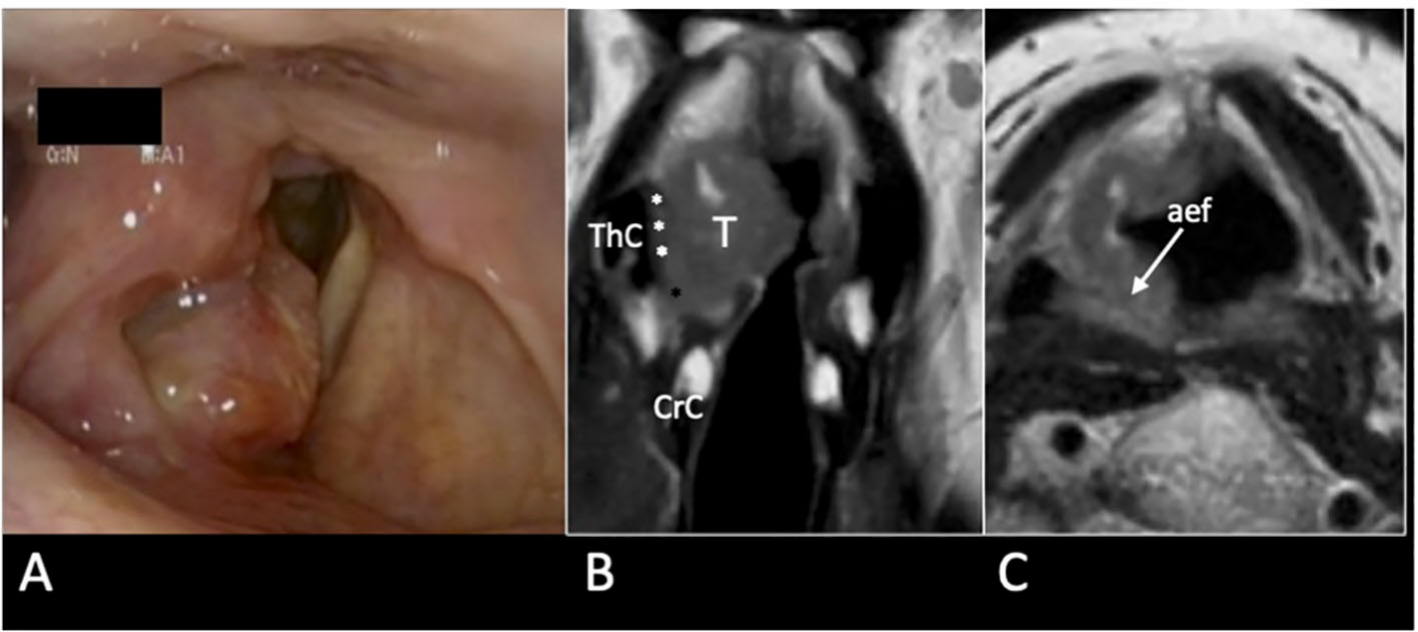
cT3 supraglottic tumour with superior paraglottic space (PGS) involvement. **(A)** Videoendoscopy shows a swelling of the right false vocal cord, an enlargement of the aryepiglottic fold, and a suspected transglottic extension. MRI T2-weighted sequence on coronal plane **(B)** demonstrates a right transglottic lesion (T) that mainly involves the supraglottic level where the tumour massively infiltrates the superior PGS (white asterisks) with a “weight” effect on the arytenoid. Inferiorly, the tumor involves the PGS at the glottic level (black asterisk). The thyroid (ThC) and the cricoid (CrC) cartilages do not show any sign of invasion. On axial plane **(C)** submucosal involvement of the right aryepiglottic fold is pointed (white arrow).

B) Transglottic evolution with possible fixation of the arytenoid: A detailed study of the joint and crico-arytenoid unit (CAU) is of paramount importance. Fortunately, it is rare for a supraglottic tumor to cause arytenoid fixation from actual massive involvement of the CAU; more frequently this is due to the so-called weight effect (**Figures 12A–C**). Such an effect can be inferred on imaging when the coronal view shows the tumor in contact with an otherwise normal arytenoid cartilage.

C) The involvement of the arytenoid with its encasement and possible infiltration: in surgical terms the lateral, posterior, and inferior limits of an OPHL type I are the lateral wall of the piriform sinus, the arytenoid, and the apex of the ventricle, respectively. When a tumor affects the superior PGS, each margin may be close or positive. Thus, modulation from OPHL type I to OPHL type I + PIR rather than OPHL type IIb ± ARY allows radical margins to be obtained.

**Figure 13:**
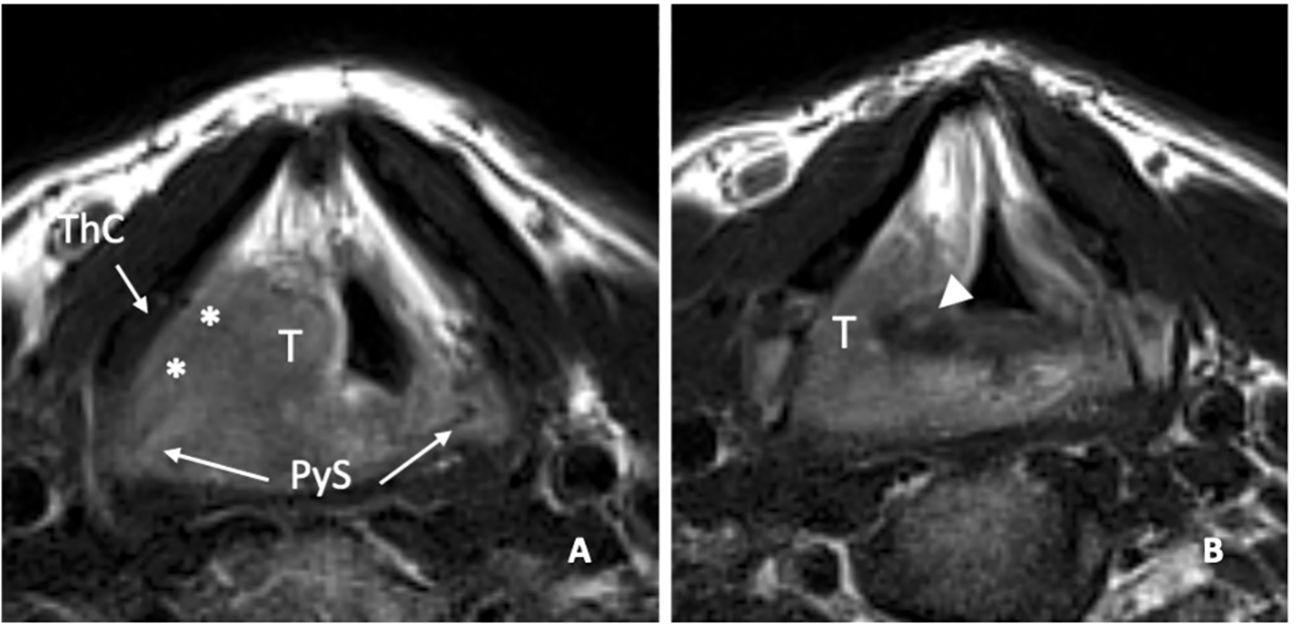
MRI T2-weighted image on axial plane shows a right supraglottic tumour (T) that massively infiltrates the superior paraglottic space (**A**, asterisks) and involves the right piriform sinus (PyS). The non-ossified thyroid cartilage (ThC) is not involved. The lesion appears indissociable from the right arytenoid (white arrowhead) that shows signal alteration with a sclerotic aspect **(B)**.

When imaging allows the exclusion of CAU infiltration, for example, in the case the arytenoid hypomobility due to a weight effect, it is possible to opt for non-surgical conservative treatment, rather than choosing partial surgery at the risk of lack of radicality (**Figure 13**).

**Figure 14:**
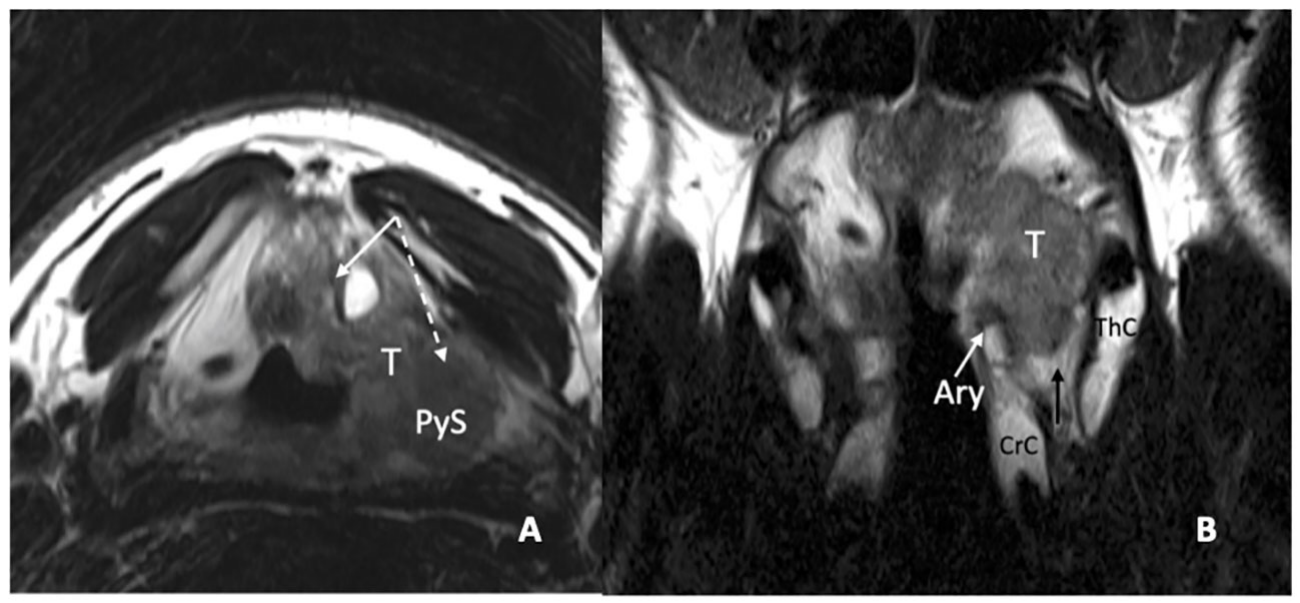
MRI T2-weighted images. Axial plane **(A)**: at supraglottic level, the lesion (T) involves the pre-epiglottic space (white arrow) and the superior paraglottic space (PGS) on the left (dashed white arrow). The ipsilateral piriform sinus (PyS) is invaded. Coronal plane **(B)**, the left transglottic tumour (T) mainly develops in the supraglottis, surrounds the ipsilateral arytenoid (Ary), partially sclerotic, and spreads towards the lower-posterior portion of the PGS, between the arytenoid and the thyroid cartilage (ThC). The inferior PGS (black arrow) is enlarged and shows high signal due to peritumoral inflammatory changes.

In a recent study, it was demonstrated that MRI, performed with a state-of-the-art technique, could predict tumoral invasion of the CAU, distinguishing it from peritumoral inflammation (22) (**Figure 14**). It must be emphasized that arytenoid sclerosis on CT is a nonspecific finding and should not be automatically interpreted as a sign of tumor infiltration.

#### 2.2.6–8 Are the inferior and posterior paraglottic spaces involved? Is the arytenoid cartilage involved?

**Figure 15:**
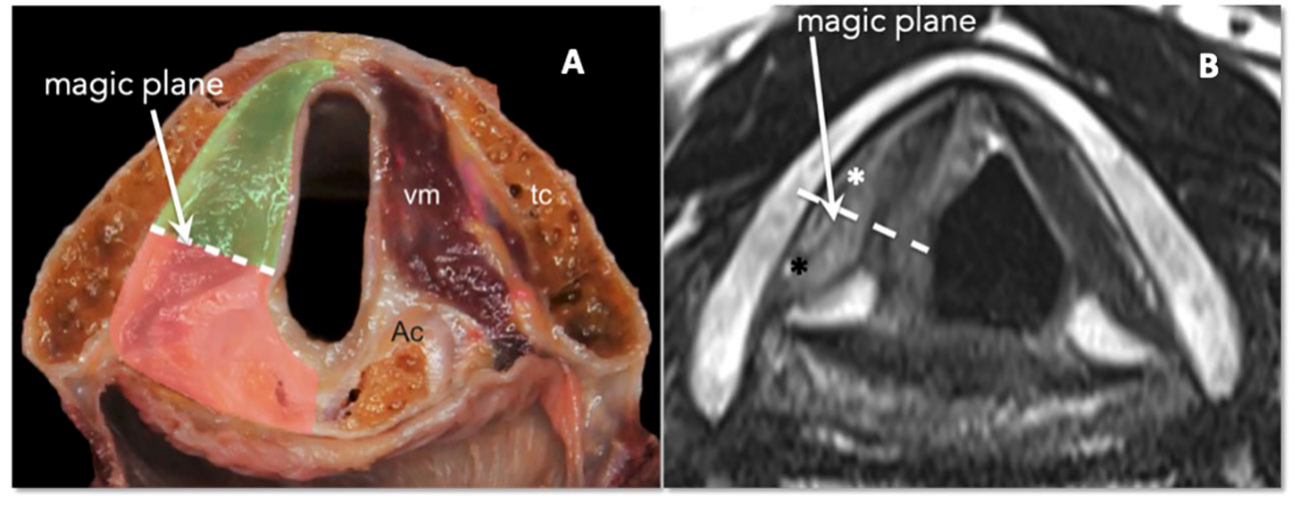
**(A)** Scheme of laryngeal compartmentalization: posterior part. **(B)** Corresponding MRI T2-weighted image on the axial plane at the glottic level shows anterior (white asterisk) and posterior PGS (black asterisk). The “magic plane” is defined drawing a line from the vocal process of the arytenoid perpendicularly to the ipsilateral thyroid lamina.

At the glottic level, tumor extension to the PGS plays a fundamental role in conditioning submucosal involvement, which represents one of the most insidious routes of spread of LC. Recent studies have introduced the concept of compartmentalization of the PGS into the anterior and posterior parts, using a conventional line called the “magic plane,” with the aim of optimizing the indications for both open and endoscopic partial surgery of the larynx (23) (**Figures 15A, B**).

The PGS below the glottic plane continues into the inferior PGS (6).

In a retrospective analysis of 17 patients affected by cT3 glottic LC with arytenoid fixation, Ravanelli et al. demonstrated that multiparametric MRI obtained with surface coils can provide an accurate definition of the tumoral involvement of the different structures contained in the posterior PGS, including the arytenoid cartilage. Combining information from T2-weighted sequences, DWI, and contrast-enhanced T1, three signal intensity patterns for each structure were defined that could be used to differentiate neoplastic infiltration, peritumoral inflammation, and weight effects (22) (**Supplementary Figures 1**, **2**).

These anatomical aspects, matched with the functional impact on vocal cord and arytenoid fixation, have demonstrated both simplicity and objectivity in their application as well as linearity in defining prognostic outcomes (23, 24).

Added to these aspects is the possible involvement of the arytenoid cartilage, one of the key elements of CAU. The controversies and limitations in demonstrating invasion or non-invasion of this cartilage are well known (25, 26). The involvement of the arytenoid must be suspected in cases of continuity of the tumor with the cartilage itself, which is often associated with complete arytenoid sclerosis (sensitive radiologic sign, although not very specific) (27).

The repercussions in terms of modulation of surgery are intuitive; in every case of even minimal transgression of the magic plane or arytenoid sclerosis in contiguity with the tumor, the modulation of surgery encompasses an enlargement of OPHL type II to the arytenoid (**Supplementary Figures 3A, B**).

Massive transgression of the magic plane, which corresponds to arytenoid fixation and, as a rule, involvement of the inferior PGS, is an absolute contraindication to OPHL type II + ARY, resulting in the need for OPHL type III extended to the CAU or a total laryngectomy (**Supplementary Figures 4A, B**).

#### 2.2.9–10 Are the crico-arytenoid joint and the thyro-crico-arytenoid space involved?

Arytenoid fixation can be linked to various causes, and as has been well demonstrated in various studies, it usually assumes negative prognostic significance in cases of open partial surgery (13, 28). Among the causes of arytenoid fixation, direct invasion of the cricoarytenoid joint (CAJ) and thyro-crico-arytenoid space (TCAS) certainly represents the worst eventualities since they carry: A) in the first case, the absolute impossibility of radically resecting the tumor with an OPHL type II and the need for an OPHL type III + CAU; and B) in the second case, the tendency of the tumor to spread up to the submucosa of the hypopharynx at the level of the piriform sinus, thus contraindicating any kind of OPHL. On both CT and MRI, the CAJ is best evaluated in the axial plane (6).The assessment of TCAS is more accurately performed using MRI, and the prescribed planes are axial and coronal (5). The symmetry of TCASs should always be checked carefully, and when an abnormally expanded TCAS is observed on axial images, the challenge is to distinguish tumor invasion from edema. Once again, MRI DWI sequences are pivotal: edema shows unrestricted diffusion, while the tumor restricts water diffusivity. On CT, edema shows lower density values than tumors, with no or minimal mass effects.

In a recent study, Succo et al. attempted to systematize the indications for OPHLs, even in cases with arytenoid fixation, by distinguishing four subcategories based on whether the CAJ, posterior PGS, spreading toward the inferior PGS, or demonstrable subglottis (29).

In subcategory III, characterized by involvement of the CAJ, posterior PGS, and subglottis, the results of loco-regional control of OPHL type III are still satisfactory (**Supplementary Figures 5A, B**, **6A, B**), whereas in subcategory IV, characterized by massive involvement of the TCAS, no OPHL can be safely carried out (**Supplementary Figures 7**, **8A–C**).

#### 2.2.11 Is the anterior commissure involved?

The involvement of the anterior commissure (AC) in the horizontal rather than vertical plane is different when it comes to providing indications for an OPHL.

Horizontal AC involvement by a glottic tumor plays a less relevant role in planning an OPHL than a TOLMS procedure or vertical partial laryngectomy (30).

In fact, the entire AC is included in the OPHL type II and III specimens. An accurate radiological study of this subsite, however, is always necessary (**Supplementary Figure 9**) since it may represent a pathway for extra-laryngeal extension through the thyroid cartilage or along its inferior border (31, 32). Wu and colleagues demonstrated a higher accuracy of MRI as compared to CT (88.5% and 57.7%, respectively) in the assessment of AC tumoral involvement (33).

The axial plane is the best to assess the horizontal involvement of the AC; a thickening of >1 mm of the AC is defined as abnormal. Correct orientation of the axial plane, parallel to the true vocal folds, is a paramount technical aspect for the correct interpretation of radiological findings.

Vertical transcommissural tumor extension requires assessment of the sagittal plane, which provides a panoramic demonstration of tumor relationships with the thyroid notch, petiole of the epiglottis, PES, and subglottis (6). This can occur in cases of supraglottic tumors extending to the glottis through anterior transglottic extension (21) or pure commissural lesions with upward (infrapetiolar) or downward extension (34). In the first case, assessment of the glottic involvement determined an absolute contraindication to OPHL type I, which can be extensively modulated with great safety and expanded up to an OPHL type IIb and, if needed, to an OPHL type IIIb with removal of the anterior cricoid arch up to the first tracheal ring (**Supplementary Figures 10A, B**). In the second case, even if the AC is completely included in the specimen of OPHL types II and III, it is of paramount importance to know in advance the possible extra-laryngeal spread. Minimal extra-laryngeal extension is not incompatible with performing an OPHL if the resection of the pre-laryngeal structures (strap muscles, thyroid isthmus, pyramidal lobe, and central compartment lymph nodes) is the same as that of total laryngectomy (19).

#### 2.2.12 Is the posterior commissure involved?

Involvement of the posterior commissure represents a clear contraindication for any type of OPHL. It is an infrequent but sometimes subtle extension that can be difficult to assess during an endoscopic workup. During direct microlaryngoscopy, the view of the posterior commissure is masked by the presence of the orotracheal tube. For a correct clinical evaluation of the posterior border of the lesion, it is necessary to remove it, to move it forward by ‘loading’ it with the laryngoscope, using a small catheter for temporary jet ventilation or, even better, an Evone flow-controlled ventilation system (35). On the other hand, imaging allows a very precise assessment of both superficial and submucosal involvement of the posterior commissure. The assessment of the posterior commissure on imaging, both on CT and MRI, is performed on the axial plane (6) (**Supplementary Figures 11A, B**). Under normal conditions, the mucosa investing in the posterior commissure should be barely visible and the air/mucosa surface should appear sharply linear. Consequently, a thickness of >1 mm, even at this level strongly suggests tumor involvement.

#### 2.2.13 Is the thyroid cartilage involved?

This element is of paramount importance in the preoperative diagnostic workup for LC, particularly if partial laryngectomy surgery is foreseen. The hyaline cartilage of which the thyroid cartilage is composed, in fact, undergoes ossification with advancing age, which can be complete or, more frequently, incomplete, with asymmetries between one side and the other, leading to awkward differential diagnosis with gaps caused by tumor invasion of the cartilage itself (36).

From a technical point of view, the thyroid cartilage is completely resected in OPHL types II–III, while only the upper half is resected in OPHL type I. In the case of cartilage infiltration with medullary involvement, the latter must be considered contraindicated due to the high recurrence rate occurring if thyroid cartilage resection is not complete. Through invasion of the thyroid cartilage, LC was classified as T4a (**Supplementary Figure 12**). Controversy persists as to whether OPHL is indicated in cT4a tumors. In this case, imaging is of fundamental importance because it allows differentiation within this category of tumors with low-volume extra-laryngeal extension from those with gross extra-laryngeal spread.

The limitations of CT in the evaluation of cartilage invasion are well known. Sclerosis alone does not allow distinguishing neoplastic involvement from inflammatory changes, mainly due to tumor proximity, with consequent low specificity values (40% for thyroid cartilage) (5, 37). On the other hand, erosion, lysis, and extra-laryngeal spread show low sensitivity; in particular, the density values of neoplastic tissue and non-ossified cartilage are quite similar, which makes the interpretation of radiological findings challenging (5). In addition, non-ossified cartilage has a homogenous density that is similar to that of the tumor.

To overcome the limitations of standard CT, radiologists can choose between two solutions: dual-energy CT (DECT) and MR. DECT is a technique that uses two X-ray beams with different voltages, allowing the reconstruction of synthetic monoenergetic images. In high-voltage images, tumor contrast enhancement is minimized while the density of non-ossified cartilage is enhanced, which increases the contrast between the tumor and cartilage, allowing improved discrimination (38, 39).

The high intrinsic tissue contrast resolution of MRI, enhanced by a multiparametric approach, may overcome the main limitations of CT, especially in improving the detection of intra-cartilaginous tumoral involvement in non-ossified cartilage (8). MRI criteria for cartilage invasion were redefined by Becker et al. as follows: the cartilage should be reported as invaded when its T2 and contrast-enhanced T1 signals are similar to those of the adjacent tumor, and when restricted diffusion is associated (5). These “new criteria” improve the specificity (75% for the thyroid cartilage) compared to the old criteria, while maintaining high sensitivity and negative predictive value (5, 40, 41) (**Supplementary Figures 13A, B**).

Recent data from Succo et al. showed that the results of OPHL types II and III are still flattering in the former and are clearly worse in the latter (19). Systematic resection of the pre-laryngeal anatomical structures is sufficient to guarantee good radicality when the tumor protrudes slightly outside the laryngeal framework.

In terms of oncologic outcomes, involvement of the framework is a significant prognostic factor for locoregional extra-laryngeal recurrence; hence, the need for systematic dissection of the central compartment of the neck (42).

#### 2.2.14-15 Are the subglottis and cricothyroid space involved? Does the tumor pass through the cricothyroid membrane?

Subglottic tumor spreading, when contained within the elastic cone, can be too subtle to be assessed endoscopically. In this case, the contribution of proper radiological imaging is crucial because of its impact on the correct planning of the most adequate type of surgery (17).

There is no visible anatomic landmark to indicate the anatomical transition from the glottis to the subglottis; conventionally, the upper limit of the subglottis is 1 cm below the glottic level. The subglottic mucosa is barely perceptible; thickness >1 mm should be interpreted again with suspicion. There is no preferred plane for subglottic assessment during imaging, being its choice is mainly dictated by the specific site of the lesion and its possible ways of spreading.

MRI provides a more precise definition of cricothyroid membrane involvement, which acts as a potential pathway for extra-laryngeal spread (17) (**Supplementary Figures 14**, **15A, B**). High-quality axial images can display the median cricothyroid ligament while, the membrane, subtle and discontinuous, is laterally not visible. A signal abnormality seen in the soft tissues anterior to the line of the membrane suggests four possible differential diagnoses: alongside tumor infiltration and peritumoral inflammation, pre-laryngeal (Delphian) lymph nodes, and pyramidal lobes of the thyroid gland should also be considered.

The resistance offered by the elastic cone tends to favor the progression of the tumor into the cricothyroid space and thus extra-laryngeal spreading, through the cricothyroid muscle and cricothyroid membrane. In this case, while generally considering the good results of OPHL in low-volume pT4a tumors, a different condition must be highlighted in comparison to the extra-laryngeal extension through the thyroid cartilage.

In fact, the tumor engagement of the cricothyroid space and the anatomical structures contained herein entails a clinical condition comparable to extra-laryngeal extension, even when this is not clearly evident. In particular, the extreme lateral proximity between the inferior border of the thyroid and the superior border of the cricoid cartilage always determines the necessity, even when there is no direct involvement of the latter, to perform an OPHL type III when imaging the tumor appears to engage the cricothyroid space or minimally involve the cricothyroid membrane. This is possible because the larynx tolerates tumor-free margins of 4 mm–5 mm. In fact, OPHLs type II would result in these cases being too at risk for close/positive margins.

#### 2.2.16 Is the cricoid cartilage involved?

The importance of detecting any invasion/contiguity of the cricoid cartilage by the tumor during the diagnostic workup is intuitive because this occurrence contraindicates the execution of any OPHL type II with the need to extend the resection to an OPHL type III when not to a total laryngectomy (**Supplementary Figures 16A, B**).

The volumetric narrowness of the cricothyroid space determines a certain degree of tumor proximity/contiguity with the cricoid whenever the lesion involves the inferior PGS and the elastic cone medially contains the neoplastic subglottic extension.

The CT criteria for cricoid involvement include sclerosis (which is non-specific) and erosion/lysis with extra-laryngeal extension (both with high positive predictive value) (5). MRI improves diagnostic accuracy in the identification of cricoid abnormalities, thus avoiding CT-related diagnostic pitfalls (5, 8). The most sensitive MRI finding was represented by hypointensity of the medullary bone marrow (**Supplementary Figures 17A–D**).

Considering the potential extreme exiguity of the lower resection margin and the good oncological and functional results of OPHL type III, it is prudent to widen the resection whenever non-specific radiological signs, such as cricoid sclerosis, are present. This attitude is maintained in the event of a positive result in frozen sections of the Delphian lymph node.

#### 2.2.17 Are the strap muscles involved?

During the work-up, a delicate aspect is related to the evaluation of whether the strap muscles are affected by the T4a LC.

In fact, the strap muscles, which can be sacrificed during OPHL types II and III, anterolaterally represent the safety resection margin when dealing with a tumor minimally spreading outside the larynx, as demonstrated by Succo et al. (19) and numerous other single-institutional case studies (24, 43). Indeed, minimal tumor extension through the framework or laryngeal membranes is contained by structures such as the perichondrium of the thyroid cartilage or the membranes themselves.

Contrast enhancement of the pre-laryngeal musculature effectively determines the condition of a tumor that has already extended to the soft tissues and is therefore no longer amenable to conservative surgery. Ultrasound (US) with high-frequency linear probes could be more sensitive than both CT and MRI for assessing minor muscle infiltration.

The same applies when Delphian lymph node positivity is strongly suspected and is aggravated by capsular rupture. The detection of this condition using frozen sections may change the indication to a more extensive OPHL type III or, even better, total laryngectomy.

In fact, especially when the tumor clearly extends beyond the limits of the larynx, both the severity of the intervention and necessity for adjuvant RT require extreme caution when considering conservative indications. Assessment of the strap muscles during imaging is usually performed in the axial plane. MRI can be defined more accurately than CT, even with a small amount of tumor tissue invading the muscles (**Supplementary Figures 18A, B**).

#### 2.2.18 Are the thyroid gland and other structures beyond the larynx involved?

This information is crucial because every gross extra-laryngeal extension due to invasion of major cartilages such as the thyroid and/or cricoid represents an absolute contraindication to any type of OPHL.

The contraindication to OPHLs derives from both the impossibility of achieving safe radicality and the need for postoperative RT (with or without chemotherapy), which may nullify the functional goals of conservative surgery.

This clinical presentation is usually a contraindication for nonsurgical organ preservation. Therefore, in these cases, total laryngectomy plus postoperative (C)RT is considered the most appropriate treatment (44). Thyroid gland invasion can be equally demonstrated on US, CT, and MRI; however, the higher contrast resolution of MRI favors its role in the assessment of esophageal involvement.

The retropharyngeal space is virtual (unless expanded by edema) and the prevertebral fascia is beyond detection in cross-sectional imaging studies. Therefore, infiltration of the fascia may only be indirectly inferred when the tumor invades the muscles in the prevertebral space (17, 45).

#### 2.2.19 Is there a submucosal spreading in the trachea?

Similar to the subglottic spreading contained by the elastic cone, submucosal spreading in the trachea is difficult to assess endoscopically. The elastic cone at the level of the upper border of the cricoid cartilage is divided into two layers: an outer layer that inserts on the upper border of the cricoid and an inner layer that continues into the lamina propria of the trachea. In case of a subglottic submucosal extension, any asymmetry of the mucosal profile should always be carefully evaluated on imaging, either in the axial plane, coronal plane if the extension is lateral, or sagittal if the extension is in the anterior part. Any additional soft tissue in this region should be treated as suspicious for neoplastic involvement (**Supplementary Figure 19**). In this case, the intraoperative strategy may involve submucosal detachment of the ring and the anterior part of the cricoid plate, which makes it easy to follow the internal layer of the elastic cone and incise the mucosa at the level of the lower edge of the cricoid (**Supplementary Figures 20A, B**). This is the exact level at which a radicality check can be performed using frozen sections. If the inferior margin is free but the tumor reaches the level of the cricoid, it is advisable to modulate the resection from OPHL type II to type III to gain radicality on the cartilage.

If frozen sections show involvement of the lower mucosal margin, it is necessary to convert the operation into a total laryngectomy extended to the first tracheal rings.

#### 2.2.20 Are the cervical nodes involved with or without extra-nodal extension?

The presence or absence of metastatic involvement of cervical lymph nodes is of great importance when indicating OPHL. Historically, the presence of lateral neck metastases has never been a contraindication for OPHL type I, even when postoperative RT is deemed necessary. The glottis is usually spared with this type of surgery and, in case of radical T surgery, modern RT techniques allow shaping the fields to reduce/avoid the irradiation of the tumor bed and consequently the dysfunctional sequelae at the level of the neolarynx (chronic edema, dysphagia, etc.) (46).

The difference is the attitude regarding the indications for OPHL types II and III if metastatic nodes in the lateral or central compartments are suspected, particularly in cases of extra-nodal extension (ENE). an increased N category is associated with a higher rate of regional failure and the need for postoperative (C)RT, which, in the case of central compartment metastases, must also include the neolarynx within the irradiation field. This often results in chronic edema of the neoglottis with an inability to decannulate the patient (47). ENE itself is a negative prognostic factor that requires postoperative CRT.

Therefore, with an N category higher than cN1 and clinical–radiological suspicion of ENE, OPHL types II and III are generally contraindicated. Careful evaluation of the medial compartment is of paramount importance in all cases of subglottic or extra-laryngeal tumor spreading. Radiological workup of LC, both with CT and MRI, includes the assessment of nodal staging. The presence of suspected neck metastatic nodes, involved levels, unilaterality or bilaterality, and signs of ENE should be reported, along with the presence of metastatic invasion of the Delphian node. US has a complementary role in the characterization of nodes (providing useful imaging guidance for fine needle aspiration biopsy), as well as PET-CT, bearing in mind that imaging cannot detect microscopic disease within a node (5, 48).

## 3 Discussion

Since the introduction of the first total laryngectomy, surgeons’ efforts have been directed towards less aggressive solutions, represented by partial laryngectomies. The aim of this type of surgery is to combine oncological radicality and laryngeal preservation to guarantee good prognosis and optimal quality of life.

From the analysis of the NCCN guidelines, the most famous and followed worldwide (49), two general concepts emerge with reference to partial laryngectomies: the susceptibility to preserve the larynx (patient-related factors) and case selection, the latter considered as a classic tumor-related factor (loco-regional extension). For both evaluations, the experience of the operators comes heavily into play, which is directly proportional to the volume of treated cases.

Moreover, in this paper, we have faithfully reported a list of 20 questions (**Table 1**) that highly experienced surgeons ask to equally experienced head and neck radiologists in the fine diagnostic work-up process leading to the decision whether to perform an OPHL and, more specifically, which type of OPHL to carry out.

**Table 1:** Checklist of questions to be made to the Radiologist to plan an OPHL.

N°	Anatomical structure	Preferable imaging	Contraindications to OPHL (relative/absolute)
1	Base of tongue (BOT)	CT–MRI (sagittal plane)	Absolute contraindications to any OPHL types II–III Relative contraindication to OPHL type I + BOT
2	Pharyngo-epiglottic fold/lateral wall of piriform sinus	CT (Valsalva maneuver)	Absolute contraindications to any OPHL types II–III Relative contraindication to OPHL type I + PIR
3	Hyoid bone	CT (cortical erosion) MRI (medullary bone) (axial + sagittal plane)	Absolute contraindication to any type of OPHL
4	Epiglottis/pre-epiglottic space	CT–MRI (axial/sagittal plane)	PES involvement = absolute contraindication to any OPHL types IIa–IIIa
5	Thyrohyoid membrane	MRI	Absolute contraindication to any OPHL types IIa–IIIa
6	Superior paraglottic space	CT–MRI (axial/coronal plane)	Absolute contraindication to OPHL type I
7	Inferior/posterior paraglottic space	MRI > CT	Posterior PGS involvement = absolute contraindication to OPHL type II/relative contraindication to OPHL type II + ARY
8	Arytenoid	CT–MRI	Absolute contraindication to OPHL type IIa/IIb
9	Crico-arytenoid joint/thyro-crico-arytenoid space	MRI > CT	Absolute contraindication to any OPHL type II
10	Anterior commessure	MRI > CT (axial/sagittal plane9	Absolute contraindication to any OPHL type I
11	Posterior commessure	CT–MRI (axial plane)	Absolute contraindication to any type of OPHL
12	Thyroid cartilage	MRI > conventional CT CT dual energy > conventional CT	Absolute contraindication to any OPHL type I
13	Subglottis/cricothyroid space/cricothyroid membrane	MRI (axial plane)	Absolute contraindication to any OPHL type II
14	Cricoid cartilage	CT–MRI	Absolute contraindication to any OPHL type II
15	Strap muscles	US/CT/MRI (axial plane)	Absolute contraindication to any OPHL type I Relative contraindication to any OPHL type II-III
16	Thyroid gland	US/CT/MRI (axial plane)	Focal involvement of one lobe = relative contraindication to any OPHL types II–III Massive invasion = absolute contraindication to any type of OPHL
17	Oesophagus	MRI > CT	Absolute contraindication to any type of OPHL
18	Retropharyngeal space/prevertebral fascia	CT–MRI	Absolute contraindication to any type of OPHL
19	Trachea	MRI > CT (axial, coronal and sagittal plane)	Absolute contraindication to any type of OPHL
20	Cervical nodes (lateral compartment/central compartment)	US (in good hands) > CT an MRI PET-CT	Absolute contraindication to any OPHL type II/III if >cN1 and ENE+

The general approach is similar, but not identical, to the radiological checklist provided by the ELS for planning TOLMS (6). In fact, since this is an open partial approach that is currently mainly aimed at the treatment of intermediate or locally, albeit minimally, advanced cases, it is considered that the list of questions should examine the larynx structure-by-structure. Each of the sites and subsites involved corresponds to a precise impact on the OPHL indications, which allows the partial surgical approach to be modulated from the least to the most extensive operation without losing the aims of radicality and preservation of laryngeal function.

In cases of extension beyond the reasonable limits recognized for this type of surgery, the adoption of more radical solutions such as total laryngectomy or non-surgical treatments such as CRT should be thoroughly evaluated (44, 50).

A classification of OPHLs has been recently introduced, which consists of 12 interventions that can be modulated in a reductive or extensive manner depending on the three-dimensional extent of the tumor and the related dysfunctional effects that its removal may cause. This tool was associated with the need for even more precise preoperative planning closely linked to collaboration, almost a co-responsibility, between the surgeon and the radiologist in the delicate steps of the preoperative diagnostic work-up.

It may be objected that this strategy has always characterized the work of the experienced surgeon. From our personal point of view, this is true, but to facilitate understanding even by less experienced colleagues, a scholastic approach was adopted because of experience gained over the years.

As previously reported, the core indication of OPHLs today is represented by intermediate T-category tumors and selected locally advanced tumors.

The intermediate stage of the disease corresponds to the involvement of the visceral spaces of the larynx, while the advanced stage is related to the extra-laryngeal extension through the membranes and/or cartilaginous skeleton, as well as to the extension to the mucosa and submucosa of adjacent organs. From this point of view, the level of precision that state-of-the-art imaging can offer, combined with cutting-edge endoscopy, is very useful, as it minimizes the level of error and highlights the technical feasibility of partial laryngectomies, even with minimal margins of radicality.

To achieve such levels of diagnostic accuracy, it is necessary to optimize the sensitivity and specificity of imaging examinations to differentiate between tumor and edema/peritumoral inflammation, to detect even limited invasion of major cartilages, submucosal extension, spreading into visceral spaces, and extension across membranes. Within the CT/MRI comparison, while recognizing the role of workhorse represented by CT, it is undoubtedly true that MRI has reached levels of greater accuracy regarding the definition of some key parameters of the present checklist for the correct planning of OPHLs. Therefore, to improve the concept of the ‘selection’ of cases that can be treated by such a conservative treatment strategy and considering that the patient is often evaluated within tertiary referral centers with a CT with contrast medium already performed elsewhere, for a better definition of the involvement of certain key structures, an MRI may be requested as a further diagnostic completion.

Moreover, high diagnostic accuracy is crucial in the case of OPHL as a salvage procedure for radiorecurrent laryngeal cancer (51–55) or in cases of surgical procedures adopting unconventional approaches (lateral or hybrid approaches) (56, 57).

The main limitation of this study is that it is only the result of a review of the literature and years of experience in two tertiary reference centers for laryngeal cancer. The impact of this approach has not been evaluated based on evidence-based medical criteria.

On the other hand, the most significant strength of this expert review is that it can represent an extremely useful guide for centers wishing to undertake open horizontal partial laryngeal surgery following a methodical approach.

## 4 Conclusions

The growth in knowledge combined with technological improvements has led to a further qualitative leap in the process of selecting patients eligible for both open and transoral partial laryngeal surgical approaches.

Without prejudice to the expertise and autonomy of the individual professionals involved in such an important diagnostic work-up phase, it is believed that a joint and coordinated work between clinician, radiologist, and pathologist represents a real “circle of knowledge” based on mutual quality control and involvement, which improves the performance of the individuals and the team providing these treatments.

From this point of view, a question/answer type of process is easy to understand and certainly useful, even for less experienced colleagues who wish to become familiar with this fascinating branch of laryngeal surgery.

## Author contributions

EC: Conceptualization, Writing – original draft, Writing – review & editing, Supervision. GS: Conceptualization, Supervision, Writing – original draft, Writing – review & editing. SS: Data curation, Writing – original draft, Writing – review & editing. IB: Writing – review & editing. SC: Data curation, Writing – review & editing. MP: Writing – review & editing. GF: Writing – review & editing. GA: Data curation, Writing – review & editing. MT: Data curation, Writing – review & editing. CP: Writing – review & editing. DF: Writing – review & editing. MR: Conceptualization, Data curation, Supervision, Writing – original draft, Writing – review & editing.
