# Supplementary material for: Twenty questions from the surgeon to the radiologist to better plan an open partial horizontal laryngectomy
Source: Front Oncol. 2024 Jan 24;13:1305889. doi: 10.3389/fonc.2023.1305889 (PMC10847842; doi:10.3389/fonc.2023.1305889)
Supplement: Supplementary file 1 [file Image_1.pdf]

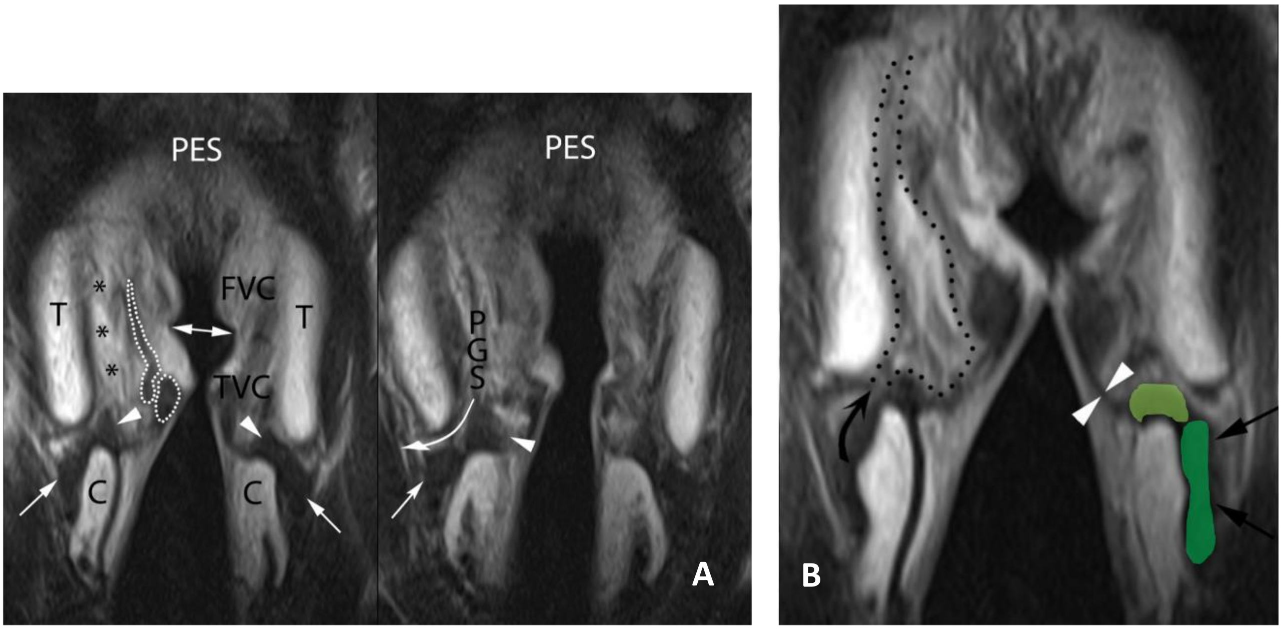

Supplementary figure 1
A) MRI T2-weighted images anatomy on coronal plane: pre-epiglottic space (PES), thyroid cartilage (T), cricoid cartilage (C), false vocal cord (FVC), true vocal cord (TVC), lateral crico-arytenoid muscle (arrowhead), crico-thyroid muscle (white arrow), paraglottic space (PGS, black asterisks); on the right image curved arrow points a potential pathway of tumour spread outside the larynx.
B) MRI T2-weighted image on coronal plane. The curved arrow points a pathway of possible extra-laryngeal tumour spread. This “gap” is normally filled with the lateral crico-arytenoid (light green) and crico-thyroid muscles (dark green). Arrowheads indicate the conus elasticus.

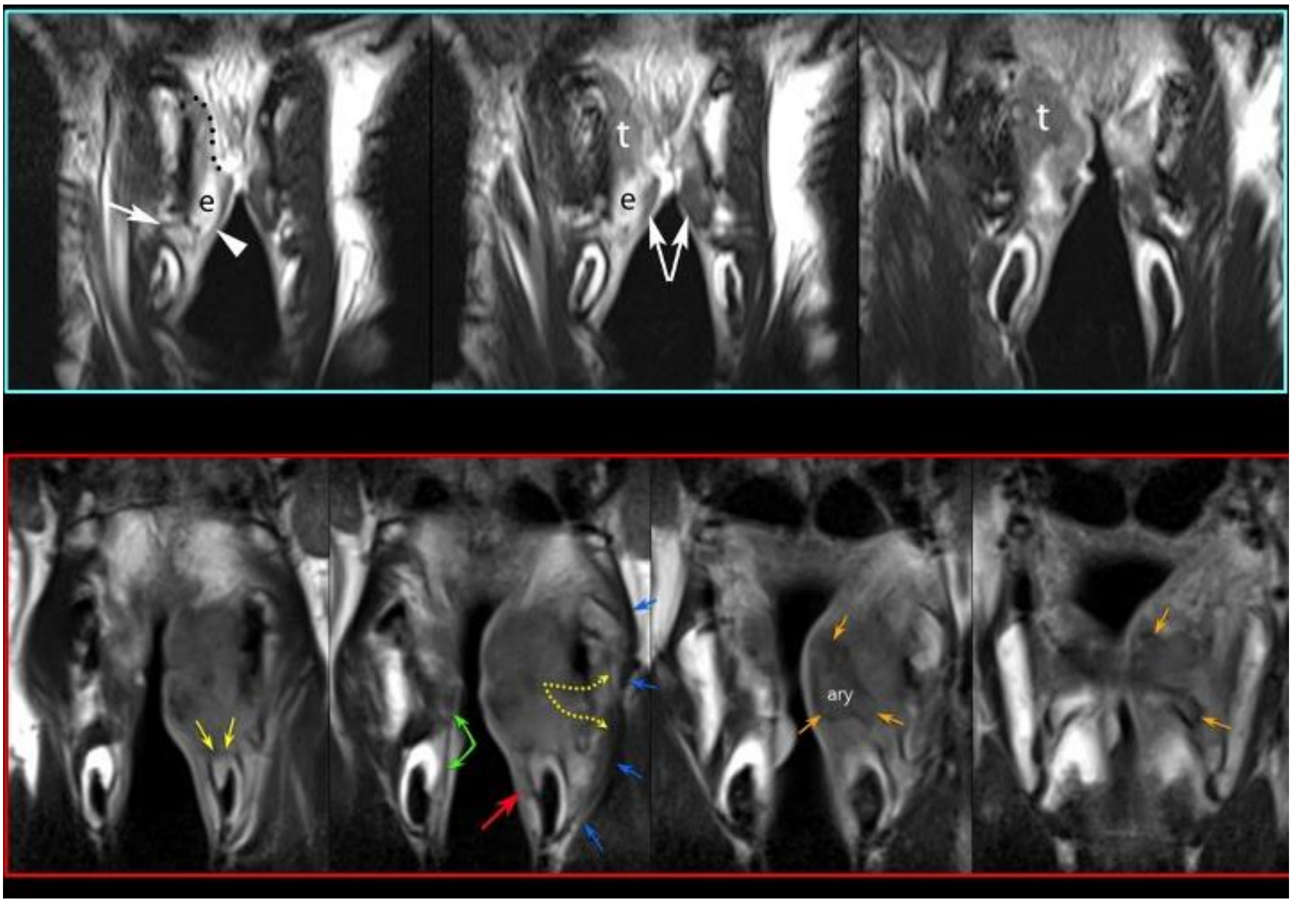

Supplementary figure 2
MRI, coronal T2-weighted images. Upper panel: a) transglottic laryngeal tumour (t) infiltrating the false and true vocal cords, mainly in the submucosal level. Oedema (e) of the right vocal and lateral cricoarytenoid muscle (white arrow); b) comparison between tumour (t) and oedema (e) and between the normal and the abnormal vocal muscle (white arrows); c) tumour in the supraglottic region. Bottom panel: a) transglottic laryngeal cancer infiltration of the left hemilarynx up to the upper margin of the cricoid lamina (yellow arrows); b) tumour infiltrates the subglottis (red arrow), the left lateral cricoarytenoid muscle, with normal appearance of the conus elasticus on the right side, oedema in the left cricothyroid muscle, no extra-laryngeal spread (blue arrows); c) submucosal spread of the tumour (presence of mucosal hyperintense lining superficially), infiltration of arytenoid; d) infiltration of the crico-arytenoid unit (orange arrows).

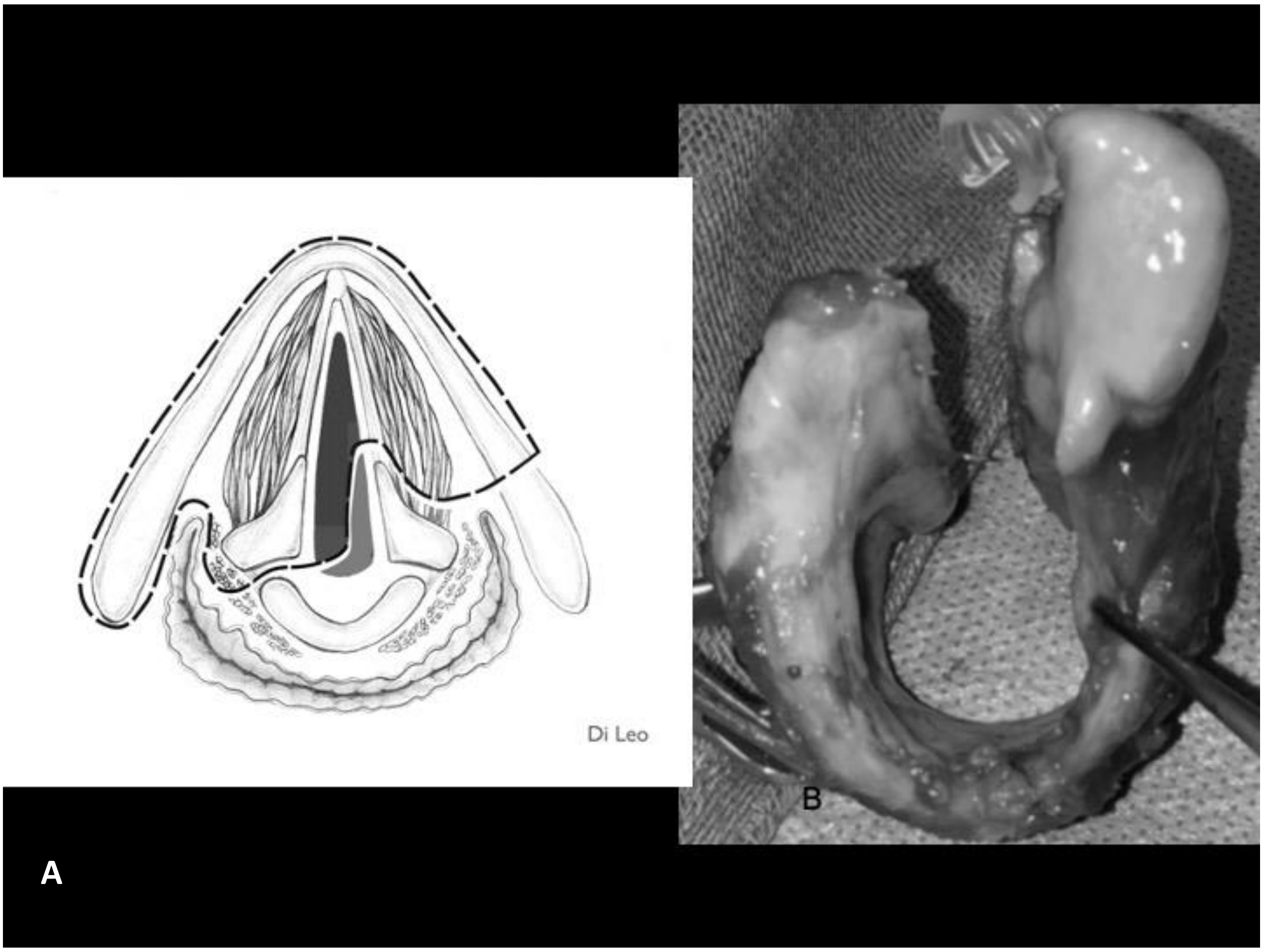

Supplementary figure 3

Scheme of a Type IIa/IIb OPHL + left ARY (axial view):

A) resection scheme.
B) the resection runs along the superior margin of the cricoid cartilage (specimen)

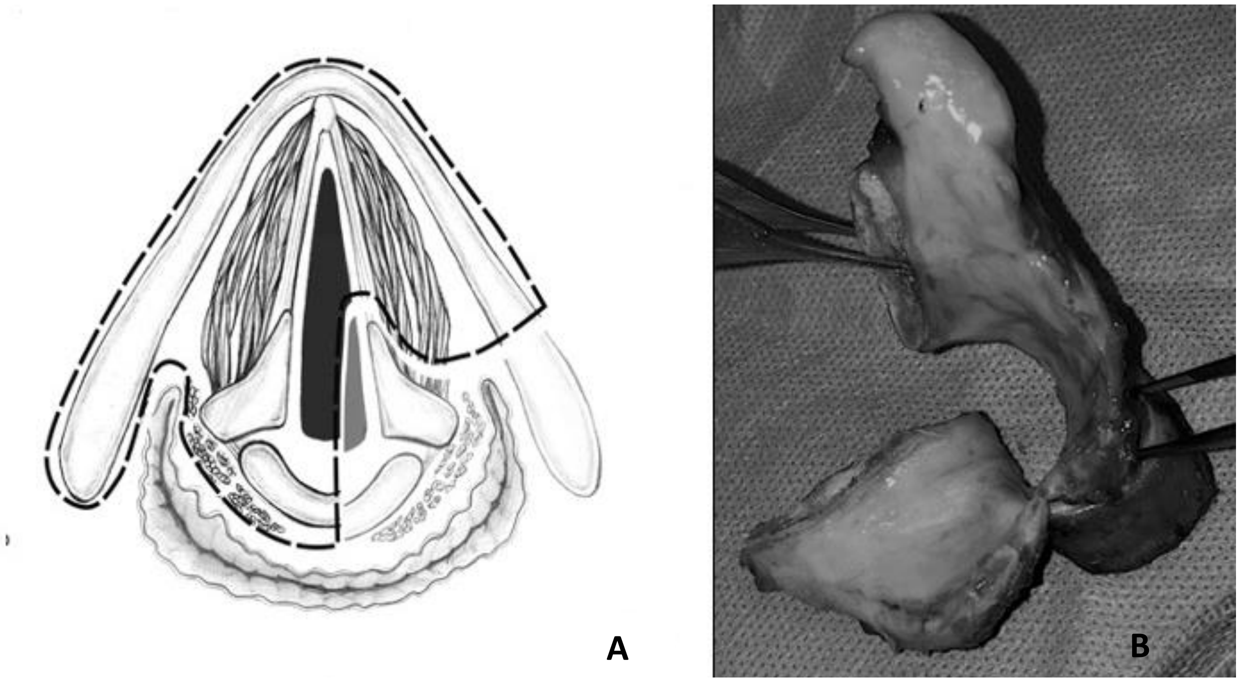

Supplementary figure 4

Scheme of a Type IIIa/IIIb OPHL + left CAU (axial view):

A) resection scheme

B) the entire left crico-arytenoid unit has been removed (specimen)

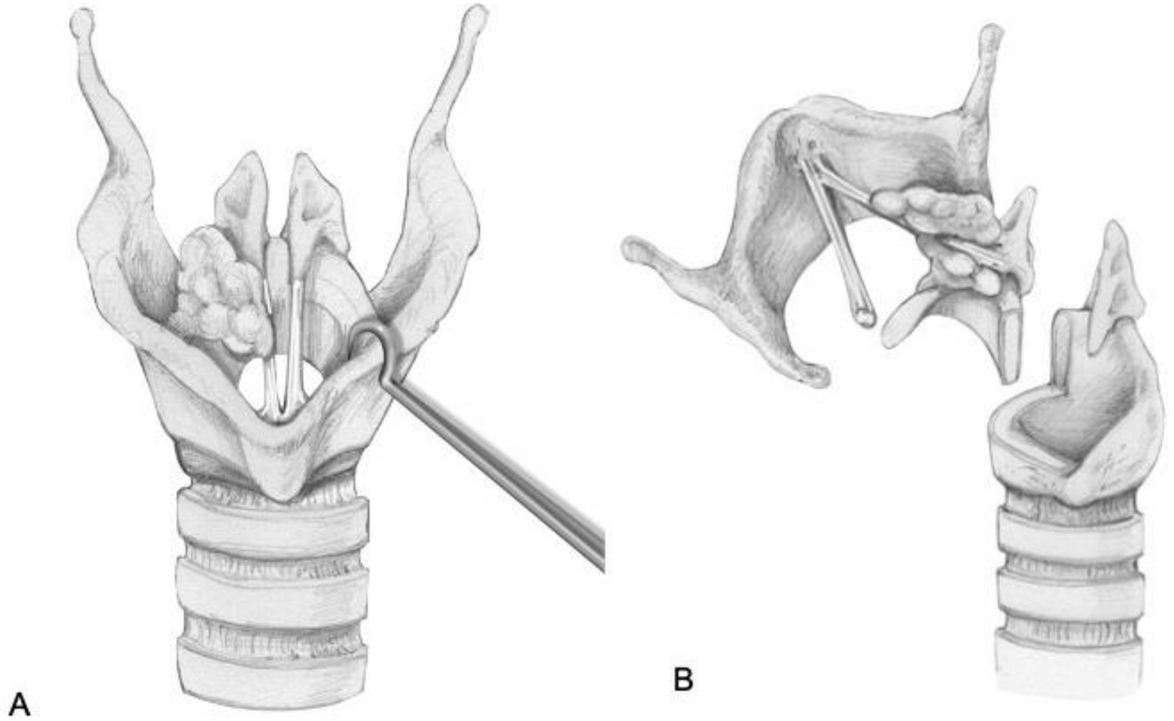

Supplementary figure 5
Scheme of a Type IIIa/IIIb OPHL + right CAU (frontal view)

A) tumour involves the inferior paraglottic space and crico-arytenoid unit
B) resection

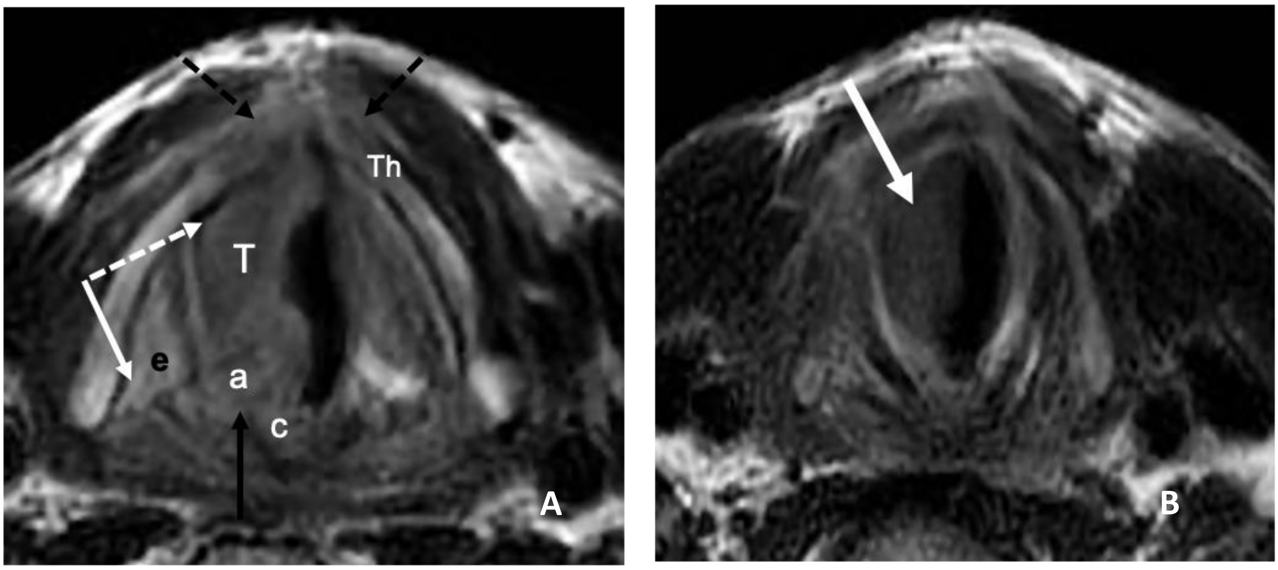

Supplementary figure 6
MRI T2-weighted images on the axial plane. At the glottic level (A) the right-sided tumour of the true vocal cord (T) invades the anterior paraglottic space (PGS, white dashed arrow) and both laminas of the thyroid cartilage (Th) with initial anterior extra-laryngeal spread making this tumour a low-volume T4 (black dashed arrows). In the right posterior PGS (white arrow), the high signal is indicative of oedema (e). Both the arytenoid (a) and cricoid (c) cartilages are involved as well as the crico-arytenoid joint (black arrow). Subglottic tumoral spread (thick white arrow) is shown (B).

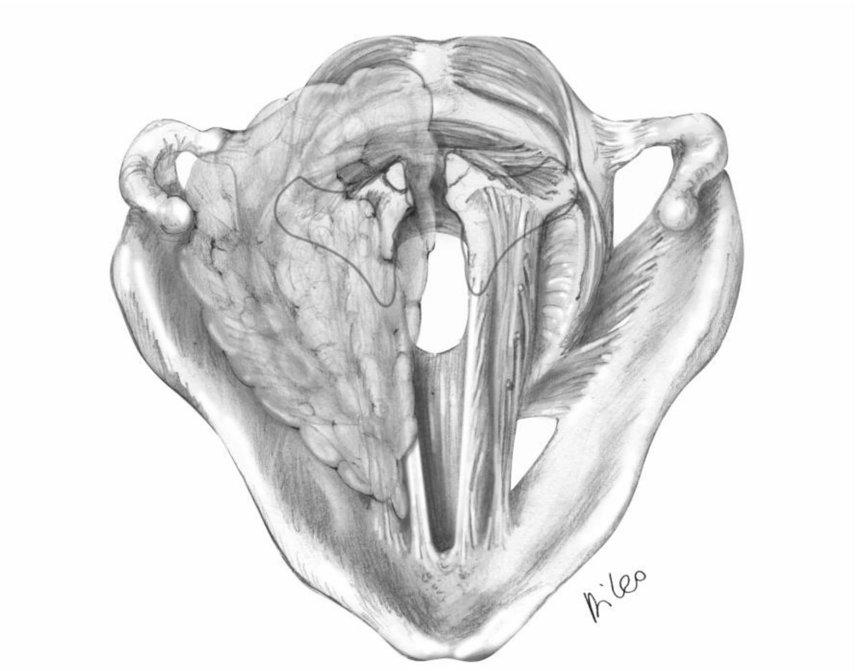

Supplementary figure 7
cT4a glottic tumor with posterior extra-laryngeal extension and thyro-crico-arytenoid space involvement.

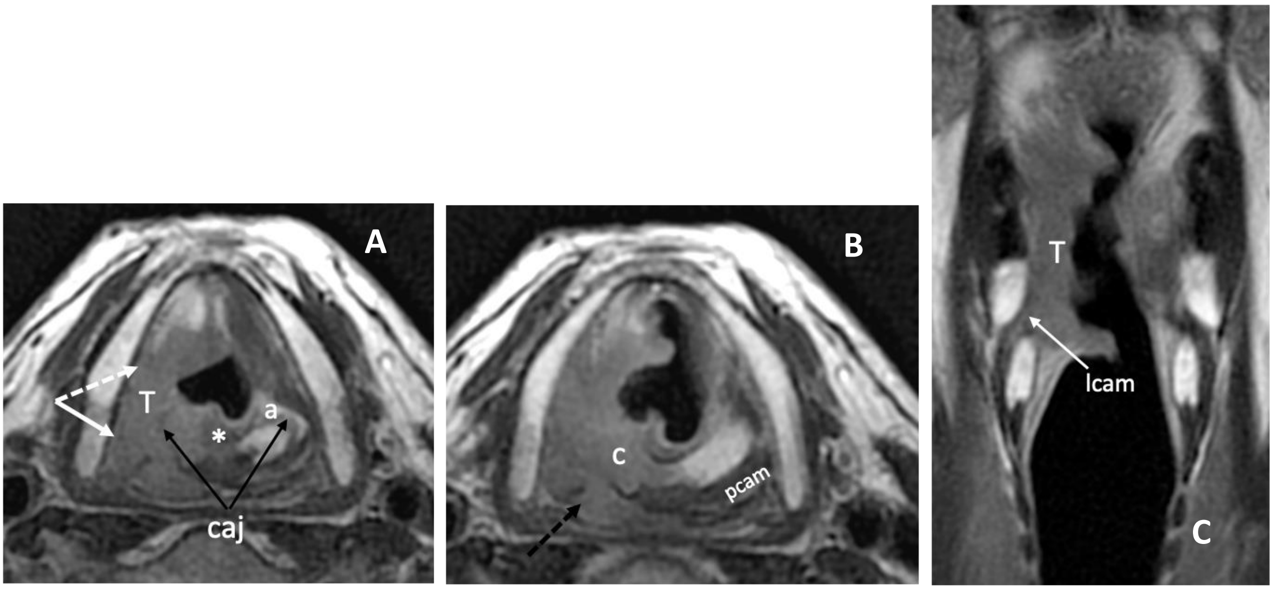

Supplementary figure 8
MRI T2-weighted images. On axial plane, at the glottic level (A) the right-sided true vocal cord tumour (T) invades the anterior (white dashed arrow) and posterior (white arrow) paraglottic space, the latter included in the thyro-crico-arytenoid space. A massive involvement of the right crico-arytenoid joint (caj) is demonstrated. The right arytenoid is completely replaced by neoplastic soft tissue, the posterior commissure (white asterisk) is infiltrated. (B) On the right side, the cricoid lamina (c) is involved as well as the posterior crico-arytenoid muscle (pcam). Submucosal spread towards the hypopharynx is indicated (black dashed arrow). The coronal plane (C) demonstrates a transglottic-subglottic extension of the tumour. The involvement of the right lateral crico-arytenoid muscle (lcam, white arrow) is clearly visible.

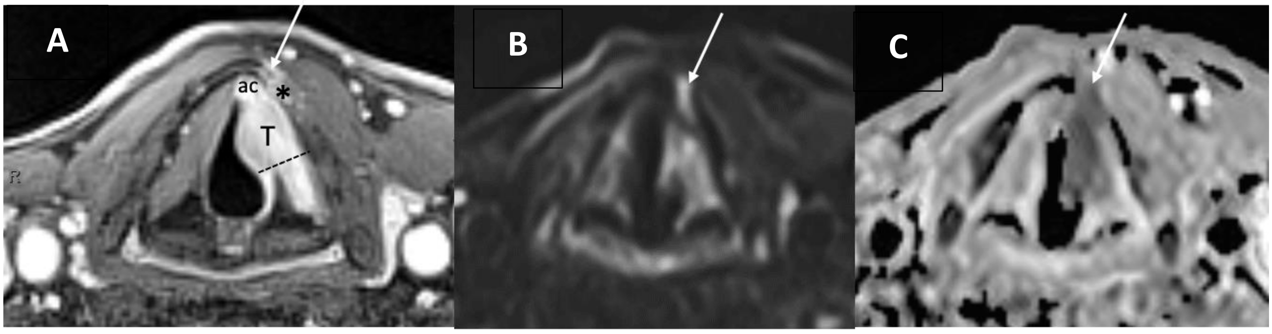

Supplementary figure 9
MRI, axial plane; T1-weighted post-contrast injection image (A), DWI b1000 (B), ADC map (C). At the glottic level (A) a left-sided true vocal cord tumour (T) involves both the anterior and posterior laryngeal compartments as defined by the “magic plane” (black dashed line). The anterior commissure (ac) is involved. The lesion infiltrates the left thyroid lamina (black asterisk) with a subtle extra-laryngeal extent (white arrow), confirmed both by DWI (B) and ADC map (C).

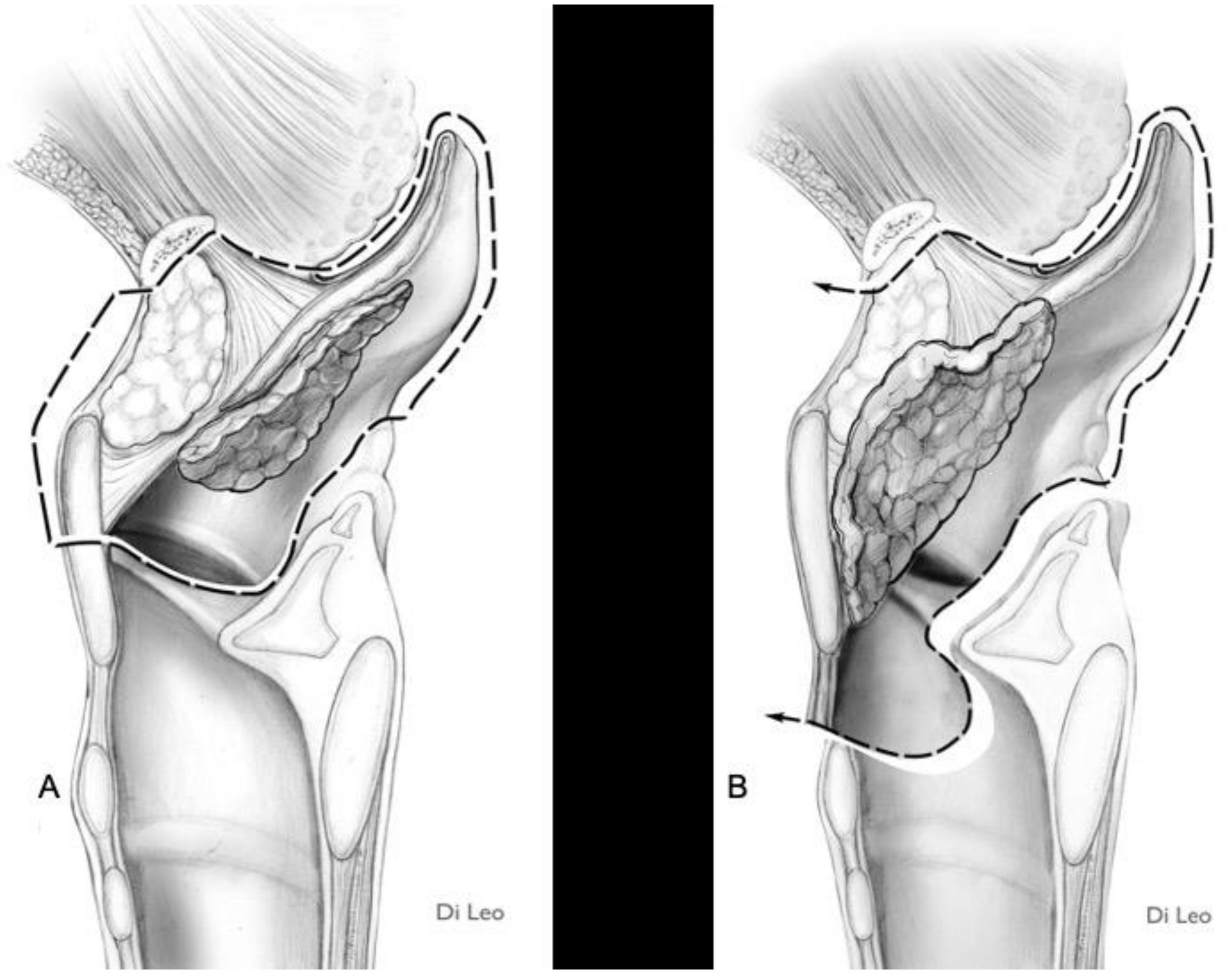

Supplementary figure 10

Comparative sagittal view of a Type I OPHL vs. a Type IIb OPHL

A) the inferior access passes through the thyroid cartilage.
B) the resection encompasses the whole thyroid cartilage.

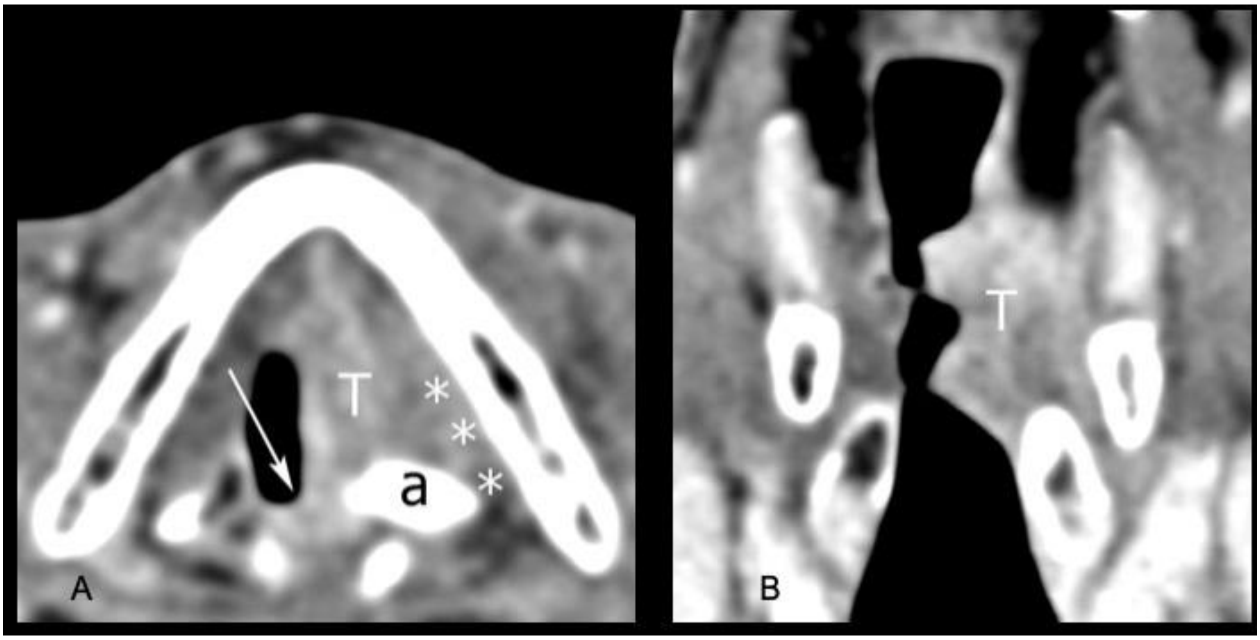

Supplementary figure 11
CT axial image (A) shows left-sided glottic tumour (T) that involves the posterior commissure (arrow). The left arytenoid (a) is sclerotic. In the posterior paraglottic space, soft tissue is detected (asterisks) but it is not possible to differentiate between tumour and peritumoral oedema. Multiplanar reconstruction on the coronal plane (B) demonstrates transglottic extension of the lesion.

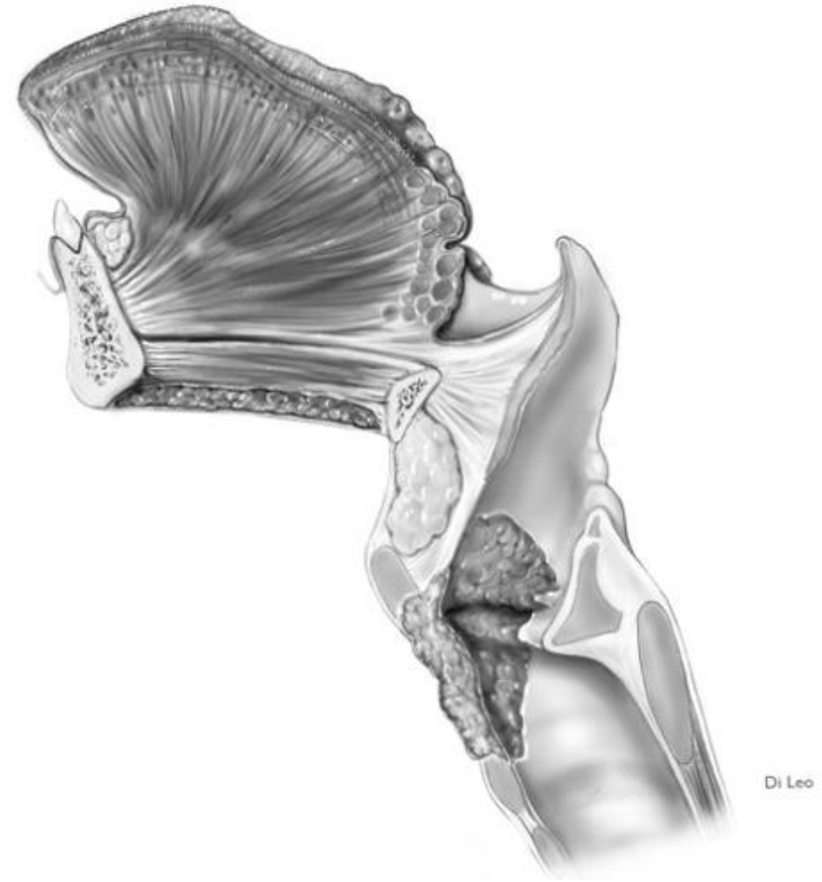

Supplementary figure 12
cT4a anterior transcommissural tumour with through and through thyroid cartilage invasion and minimal extra-laryngeal extension via the cricothyroid membrane.

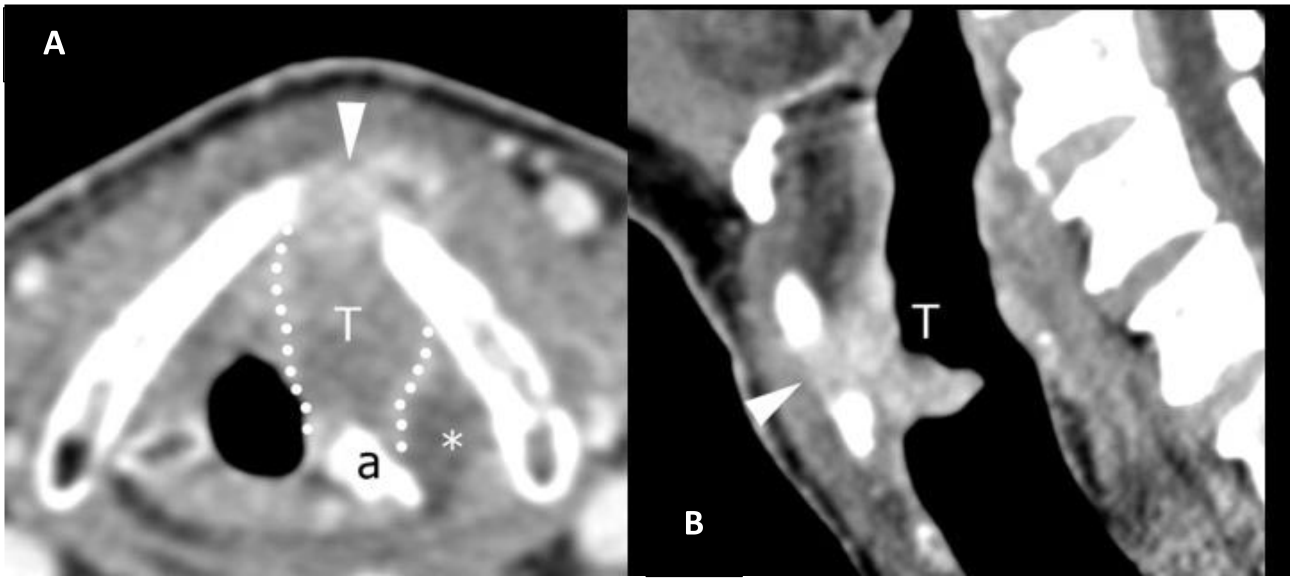

Supplementary figure 13
CT image on axial plane (A) shows a left-sided glottic tumor (T) that involves the anterior commissure. The tumor is in proximity to the left arytenoid (a), characterized by sclerosis, a non-specific finding for cartilage invasion. Low-density alteration, suggesting peritumoral oedema, is present in the enlarged posterior paraglottic space (asterisk). On sagittal plane (B) the vertical anterior transcommissural extension of the lesion is visible. Arrowheads point to the invasion of the thyroid cartilage (A, B).

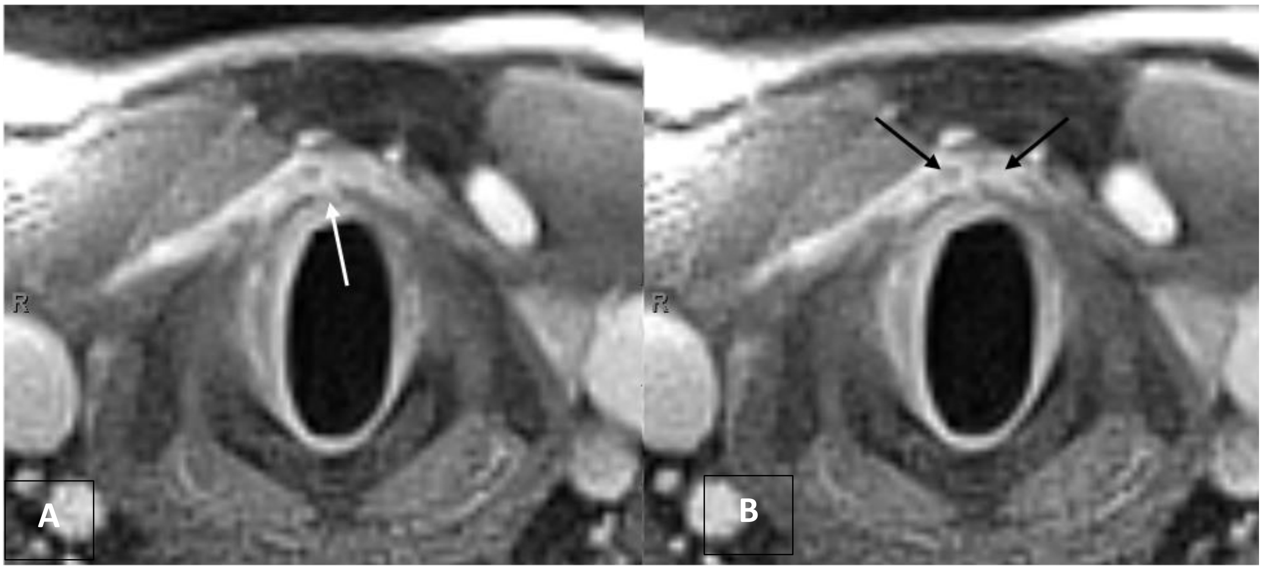

Supplementary figure 14
MRI T1-weighted images post-contrast injection on the axial plane. At the subglottic level, a subtle focal interruption (white arrow) in the cricothyroid membrane is shown (A). Black arrows point to the anterior tumoral extra-laryngeal spread (B).

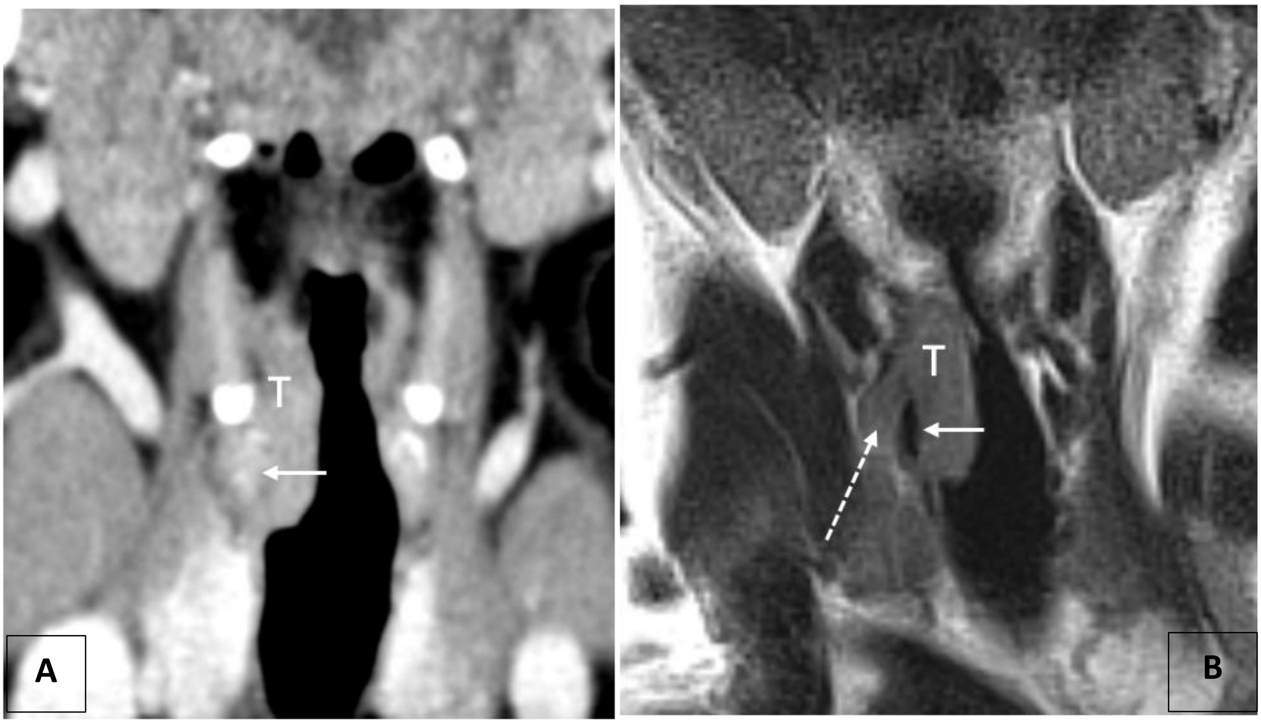

Supplementary figure 15

CT (A) and MRI T2-weighted (B) images on coronal plane demonstrate glottic-subglottic right-sided tumour (T) invading the cricoid cartilage (white arrow). MRI (B) clearly identifies extra-laryngeal spread (dashed white arrow) through the cricothyroid space.

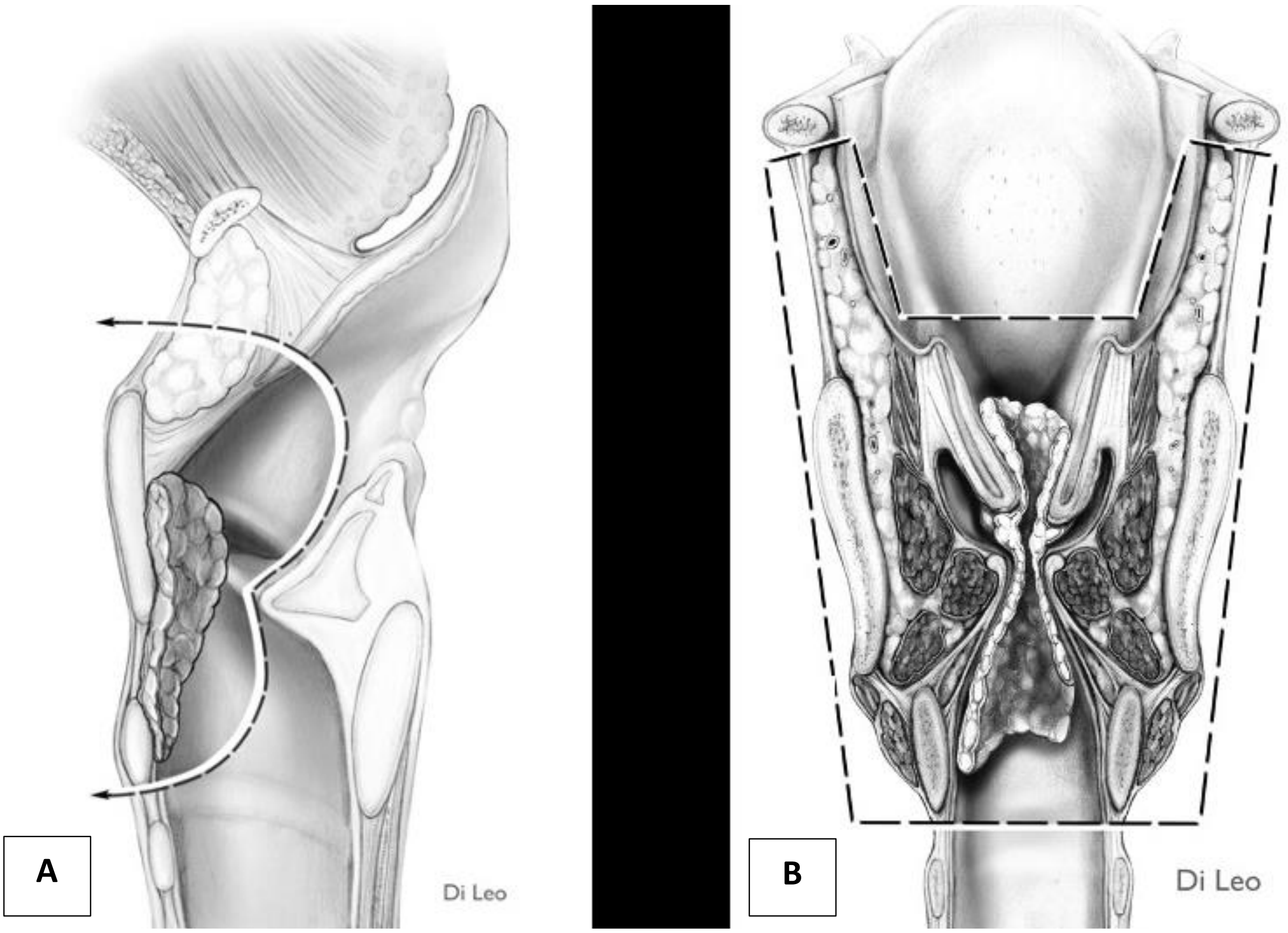

Supplementary figure 16
Type III OPHL schematic view: the inferior limit of resection encompasses the cricoid cartilage:

A) Sagittal plane
B) Coronal plane

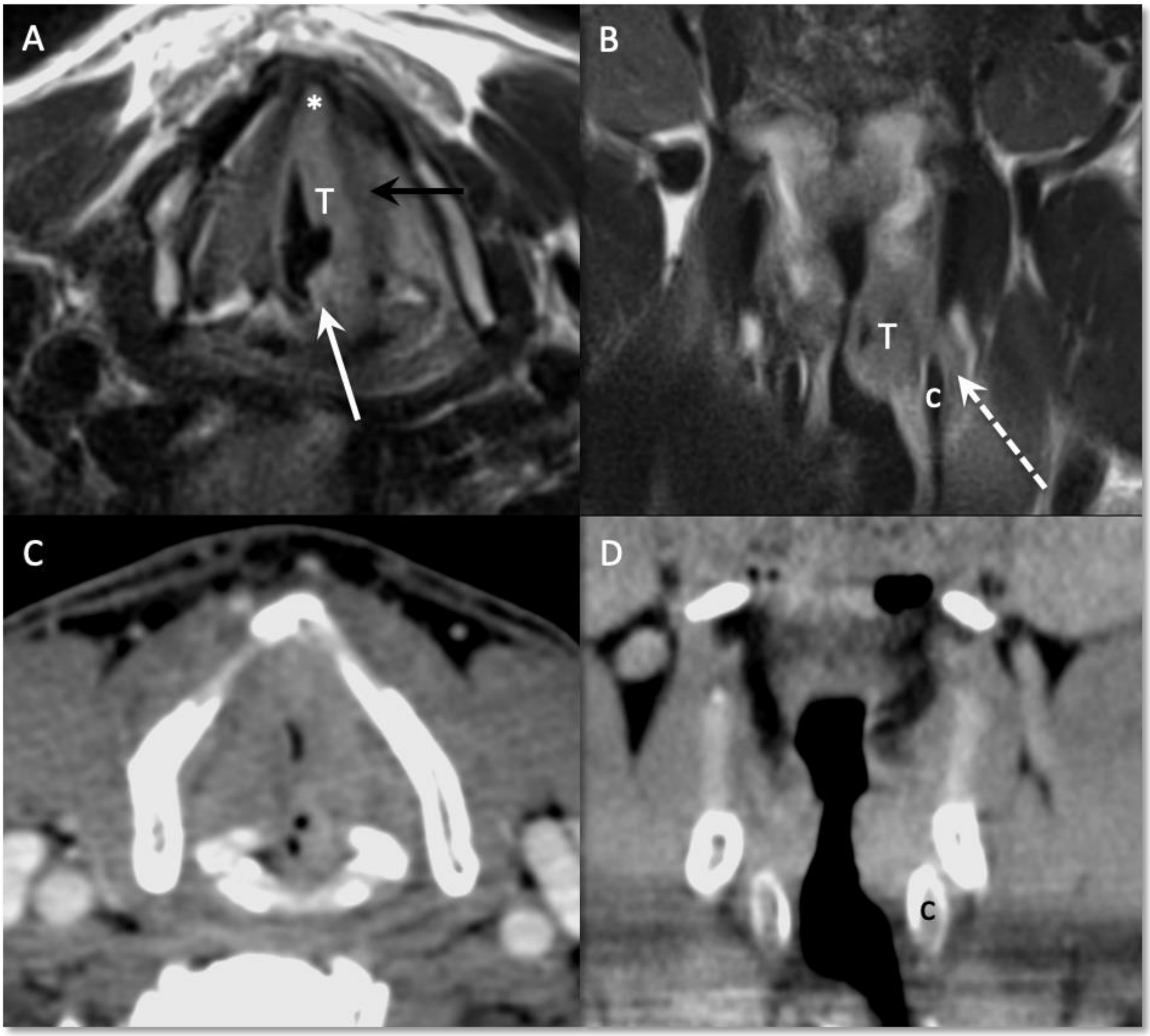

Supplementary figure 17
MRI T2-weighted image on the axial plane (A) shows a left glottic tumor (T) partially involving the thyro-arytenoid muscle, whose normal component (black arrow) is not detectable at CT (C). Anterior commissure (asterisk) and posterior commissure (white arrow) are infiltrated (A). On coronal plane both MRI (B) and CT (D) show tumour adjacent to the cricoid cartilage (c) that is not involved. MRI clearly identifies extra-laryngeal spread through the left inferior paraglottic space (B, dashed white arrow).

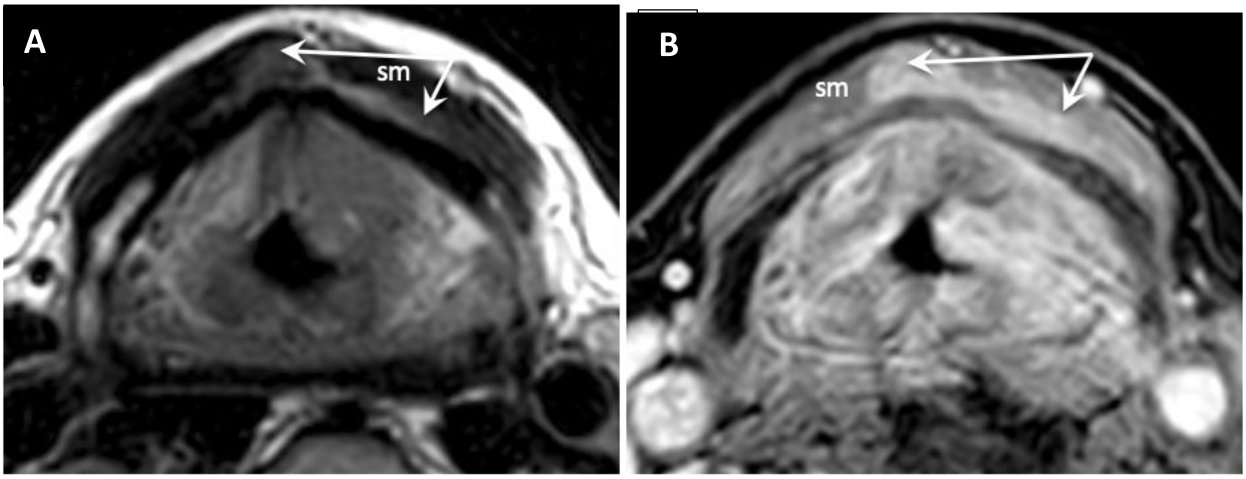

Supplementary figure 18
MRI T2-weighted on axial plane (A) and T1-weighted post-contrast injection (B) show extra-laryngeal anterior spread (arrows) of a supraglottic tumour involving the strap muscles (sm).

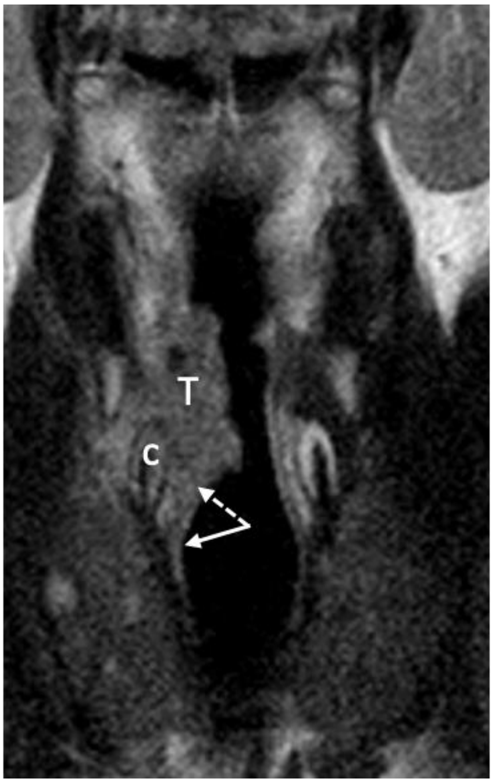

Supplementary figure 19
Coronal MRI T2-weighted image demonstrating a right-sided glottic tumour that involves the cricoid (c) and shows submucosal extent in the subglottis (dashed white arrow) and first tracheal ring (white arrow).

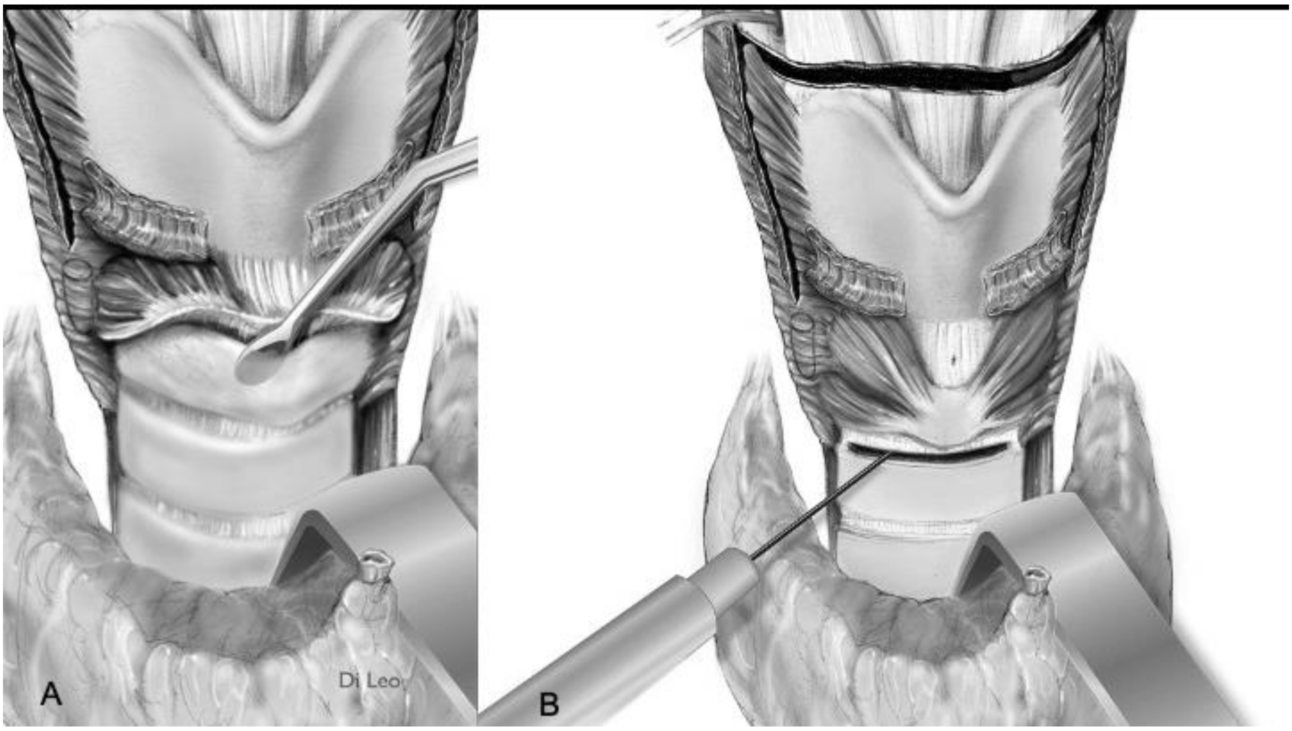

Supplementary figure 20
Technical surgical aspects:

A) Type II OPHL: the mucosa overlying the cricoid cartilage is elevated and cut at the level of the inferior margin of this cartilage.
B) Type III OPHL: both the cricoid ring and overlying mucosa are resected en-bloc up to the first tracheal ring.
